# RecJ3/4-aRNase J form a Ubl-associated nuclease complex functioning in survival against DNA damage in *Haloferax volcanii*

**DOI:** 10.1128/mbio.00852-23

**Published:** 2023-07-17

**Authors:** Huiyong Jia, Swathi Dantuluri, Shae Margulies, Victoria Smith, Rebecca Lever, Thorsten Allers, Jin Koh, Sixue Chen, Julie A. Maupin-Furlow

**Affiliations:** 1 Department of Microbiology and Cell Science, Institute of Food and Agricultural Science, University of Florida, Gainesville, Florida, USA; 2 School of Life Sciences, University of Nottingham, Nottingham, United Kingdom; 3 Proteomics and Mass Spectrometry, Interdisciplinary Center for Biotechnology Research, University of Florida, Gainesville, Florida, USA; 4 Genetics Institute, University of Florida, Gainesville, Florida, USA; 5 Department of Biology, College of Liberal Arts and Sciences, University of Florida, Gainesville, Florida, USA; Institut Pasteur, Paris, France; University of Toulouse III, Toulouse, France

**Keywords:** post-translational modification, ubiquitin, proteasomes, DNA repair, archaea

## Abstract

Nucleases are strictly regulated and often localized in the cell to avoid the uncontrolled degradation of DNA and RNA. Here, a new type of nuclease complex, composed of RecJ3, RecJ4, and aRNase J, was identified through its ATP-dependent association with the ubiquitin-like SAMP1 and AAA-ATPase Cdc48a. The complex was discovered in *Haloferax volcanii*, an archaeon lacking an RNA exosome. Genetic analysis revealed aRNase J to be essential and RecJ3, RecJ4, and Cdc48a to function in the recovery from DNA damage including genotoxic agents that generate double-strand breaks. The RecJ3:RecJ4:aRNase J complex (isolated in 2:2:1 stoichiometry) functioned primarily as a 3′−5′ exonuclease in hydrolyzing RNA and ssDNA, with the mechanism non-processive for ssDNA. aRNase J could also be purified as a homodimer that catalyzed endoribonuclease activity and, thus, was not restricted to the 5′−3′ exonuclease activity typical of aRNase J homologs. Moreover, RecJ3 and RecJ4 could be purified as a 560-kDa subcomplex in equimolar subunit ratio with nuclease activities mirroring the full RecJ3/4-aRNase J complex. These findings prompted reconstitution assays that suggested RecJ3/4 could suppress, alter, and/or outcompete the nuclease activities of aRNase J. Based on the phenotypic results, this control mechanism of aRNase J by RecJ3/4 is not necessary for cell growth but instead appears important for DNA repair.

**IMPORTANCE:**

Nucleases are critical for various cellular processes including DNA replication and repair. Here, a dynamic type of nuclease complex is newly identified in the archaeon *Haloferax volcanii*, which is missing the canonical RNA exosome. The complex, composed of RecJ3, RecJ4, and aRNase J, functions primarily as a 3′–5′ exonuclease and was discovered through its ATP-dependent association with the ubiquitin-like SAMP1 and Cdc48a. aRNase J alone forms a homodimer that has endonuclease function and, thus, is not restricted to 5′–3′ exonuclease activity typical of other aRNase J enzymes. RecJ3/4 appears to suppress, alter, and/or outcompete the nuclease activities of aRNase J. While aRNase J is essential for growth, RecJ3/4, Cdc48a, and SAMPs are important for recovery against DNA damage. These biological distinctions may correlate with the regulated nuclease activity of aRNase J in the RecJ3/4-aRNaseJ complex.

## INTRODUCTION

Nucleases are enzymes that are important in cell biology and are often strictly regulated to avoid the uncontrolled degradation of DNA and RNA. Nucleases are used in many cellular processes, including DNA replication, DNA recombination and repair, RNA maturation, RNA processing, RNA interference, nutrient regeneration, and cell death (1). Nucleases can regulate mRNA abundance in response to environmental, developmental, and metabolic cues (2, 3). They can also maintain RNA quality by preventing the damaging effects of aberrant non-coding RNA accumulation or defective mRNA translation (4). Interestingly, in addition to DNases, RNases are recently found important in DNA repair, with associated components identified in the proximity of double-stranded (ds) DNA breaks (5, 6).

RNases are generally divided into two groups: (i) endonucleases that cleave the RNA internally and (ii) exonucleases that cleave the RNA from the 3′ or 5′ ends. Some RNases have both types of activities [e.g., (7 - 9)]. These catalytic activities are controlled to ensure the proper selection and timing of the RNA species to be hydrolyzed (10). RNA degradation is regulated by various factors including mechanisms of post-transcriptional, post-translational, and *trans*-acting protein or non-coding RNA inhibitor control (10). RNases can also be associated in multisubunit complexes and/or localized in the cell to prevent the uncontrolled degradation of RNA (10).

Many archaea encode a multisubunit complex that appears central to RNA hydrolysis and shares an evolutionary relationship to the eukaryotic RNA exosome. The core of this archaeal RNA-degrading machine is composed of Rrp41 (3′−5′ exoribonuclease and poly-RNA tailing), Rrp42 (non-catalytic scaffold), Rrp4 (RNA-binding), Csl4 (RNA presentation), and DnaG (DNA primase) (11). Additional subunits include Nop5 (rRNA/tRNA 2′-*O*-methyl-transferase) (12), aRNase J, and a Ski2-like RNA helicase (13). Halophilic archaea and certain methanogens, however, do not encode an RNA exosome.

RNase J proteins are widespread in prokaryotes and key members of the β-CASP family of metallo-β-lactamases. In archaea, these enzymes function as 5′−3′ exonucleases as revealed by the study of *Pyrococcus abyssi* Pab-aRNase J (13, 14), *Thermococcus kodakarensis* Tko-aRNase J (14), *Methanolobus psychrophilus* Mpy-aRNase J (15), and *Methanocaldococcus jannaschii* Mja-aRNase J1 (16). RNA unwinding activity is also observed for Mpy-aRNase J (17). By comparison, bacterial RNase J enzymes function as 5′−3′ exo- and/or endoribonucleases (7, 18 - 23) with differences in metal ion binding, oligomerization, C-terminal extensions, and RNA interactions suggested to determine these catalytic activities (7, 18 - 24).

RecJ homologs are conserved in all domains of life and found to hydrolyze DNA and/or RNA in the 3′−5′ and/or 5′−3′ direction (25 - 27). RecJ enzymes function in DNA repair and recombination, nucleotide recycling, stress tolerance, and the final stages of chromosome duplication (28 - 33). In bacteria, such as *Escherichia coli*, RecJ is a single-stranded (ss) DNA-specific 5′−3′ exonuclease that is processive (26, 27), while other bacterial RecJ enzymes are identified that cleave dsDNA (34). Archaea have an expanded repertoire of RecJ homologs related to bacterial RecJ and eukaryal Cdc45, the latter a non-catalytic subunit of the CMG complex. The CMG complex, essential to DNA replication in eukaryotes, is composed of Cdc45, minichromosome maintenance (MCM) helicase, and GINS (from the Japanese go-ichi-ni-san meaning 5-1-2-3, after the four related subunits of the complex Sld5, Psf1, Psf2, and Psf3) (25, 35 - 37). The archaeal RecJ homolog GAN (GINS-associated nuclease) functions as a DNA/RNA exonuclease that associates with GINS and the MCM helicase (38 - 41). Moreover, the Hef-associated nuclease (HAN) (25) is an archaeal RecJ homolog that interacts with Hef, an endonuclease that cleaves DNA with various branched structures (42). Hef has an intrinsically disordered region (IDR) that is needed for association with HAN and can bind other proteins including PCNA1 (37).

**Fig 1:**
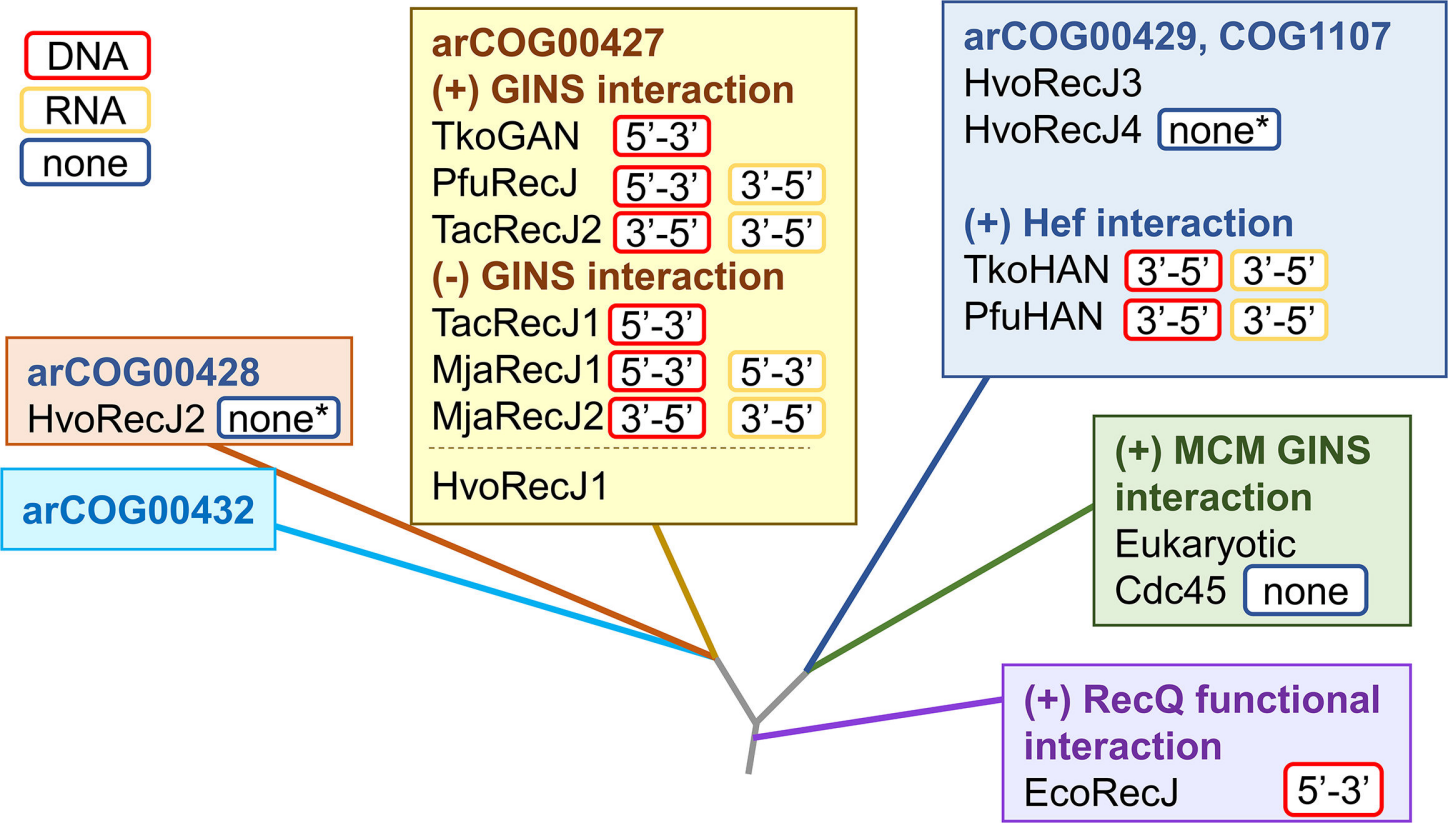
Phylogenetic relationship, nucleotidase activities, and protein partners of RecJ homologs. Figures from references (35) (phylogentic relationship) and (25) (display) were merged and modified to include HvoRecJ1-4 and the functional interaction of EcoRecJ with RecQ helicase (36). *none, presumed based on the absence of catalytic active site residues. Hvo, *H. volcanii*; Tko, *Thermococcus kodakarensis*; Pfu, *Pyrococcus furiosus*; Tac, *Thermoplasma acidophilum*; Mja, *Methanocaldococcus jannaschii*; Eco, *Escherichia coli*.

*Haloferax volcanii*, the model archaeon of this study, has an aRNase J and four RecJ (RecJ1-4) homologs that cluster into distinct arCOG groups (Fig. 1) (35). RecJ1 of arCOG00427 is closely related to GANs. The other GAN relative, RecJ2, clusters to the arCOG00428 group distinct to the members of the *Halobacteria* and *Nanoarchaeota* and is predicted to include inactive nucleases. RecJ3 and RecJ4 are members of the HAN-containing arCOG00429 group, with RecJ4 missing the conserved active site residues. An *H. volcanii ΔrecJ3* mutant is characterized and found to be impaired in survival against methyl methanesulfonate (43), a DNA alkylating agent suggested to induce stalled replication forks (44). Based on these features, *H. volcanii* RecJ3 is annotated as HAN (43) but has yet to be characterized at the biochemical level.

Post-translational modification (PTM) systems regulate mRNA turnover and surveillance as well as DNA replication and repair (45 - 48). PTMs, such as phosphorylation and the tagging of proteins with ubiquitin (Ub) or Ub-related proteins (e.g., SUMO), are important in orchestrating the progression of protein factors in eukaryotic DNA repair pathways (6, 46, 47). Proteasomes, multisubunit complexes that recognize Ub-tagged proteins, localize to sites of DNA breaks and are required for effective mRNA surveillance and protein turnover in eukaryotes (45). A Ub-like (Ubl) protein modification system is identified in archaea (49 - 53) but has yet to be associated with DNA repair/recombination or RNA degradation pathways.

Here, a new type of nuclease complex composed of RecJ3, RecJ4, and aRNase J is reported in *H. volcanii*, an archaeon lacking an RNA exosome. This complex was identified based on its transient ATP-dependent association with the Ubl SAMP1 and AAA-ATPase Cdc48a. The primary activity of the complex was identified as a 3′−5′ exonuclease against RNA and ssDNA. Further dissection of the complex revealed RecJ3/4 to associate in 1:1 molar ratio as a subcomplex with 3′−5′ exonuclease activity and aRNase J to form a homodimer. The aRNase J alone was found to have an endoribonuclease activity not previously observed for other aRNase J homologs. Reconstitution of the aRNase J with RecJ3/4 resulted in a restriction to primarily 3′−5′ exonuclease activity. Phenotypic analysis of mutant strains revealed aRNase J to be essential, while Cdc48a, RecJ3, and RecJ4 were found important for cellular recovery from DNA-damaging agents including those that introduce double-strand breaks (DSBs). These results suggest that RecJ3 and RecJ4 are ssociated with DNA repair and regulate the nuclease activities of aRNase J.

## RESULTS

### Cdc48a, aRNase J, and RecJ3/4 are associated with Ubl binding in *H. volcanii*

**Fig 2:**
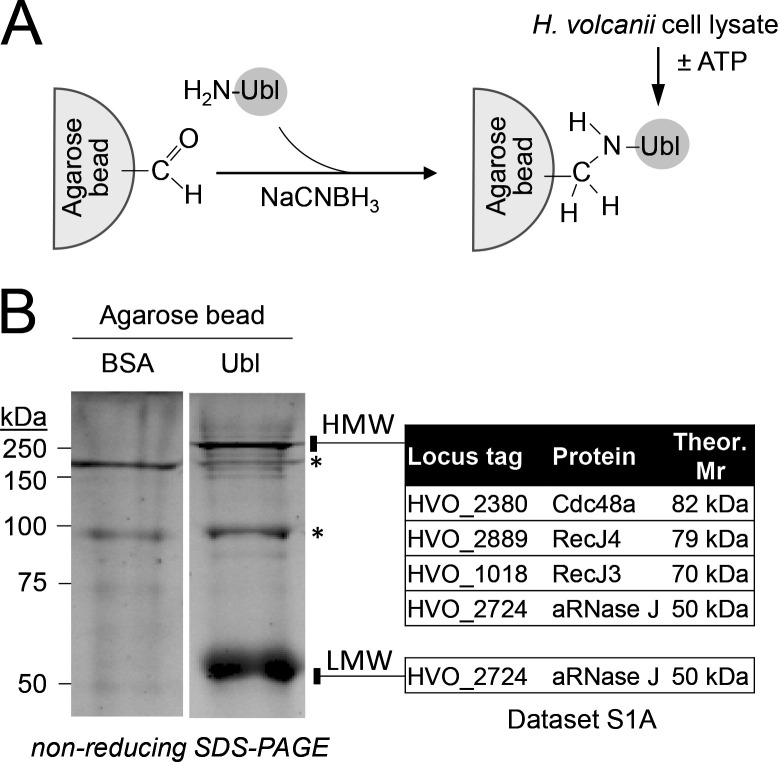
Ubl interactome of *H. volcanii* includes Cdc48a, RecJ3/4, and aRNase J. (A) Strategy to isolate *H. volcanii* proteins that bind agarose beads charged with monomeric Ubl (vs BSA) as bait. Cell lysate was from the culture of *H. volcanii* NH02-pJAM957. (B) Left, non-reducing SDS-PAGE of *H. volcanii* proteins that bound BSA- (control) vs Ubl-decorated beads in the presence of ATP as indicated. Gels were stained with SYPRO Ruby. Vertical bar on the right of gel, HMW and LMW regions of gel excised from BSA- and Ubl-associated lanes for LC-MS/MS analysis. Right, proteins were identified at >99.9% probability and <0.1% false discovery rate in the Ubl (vs BSA) samples (see Data set S1A). Similar trends were observed in independent experiments. ATP was required for this association. Theor. Mr, theoretical molecular mass based on genome sequence. Input, equal concentration and volume of cell lyate with * indicating common non-specific proteins to the Ubl and BSA control samples.

To identify Ubl interaction networks in archaea, the *H. volcanii* Ubl SAMP1 was covalently linked to agarose beads and used as bait to purify interacting proteins from *H. volcanii* cell lysate (Fig. 2A). Prior to lysis, the cells were grown to stationary phase in a medium supplemented with dimethylsulfoxide (DMSO), a condition known to stimulate Ubl-conjugate formation (54). Proteins that bound to the Ubl beads were separated by non-reducing SDS-PAGE, visualized by SYPRO Ruby staining, and identified by liquid chromatography tandem mass spectrometry (LC-MS/MS) analysis. Two major protein bands that bound the Ubl SAMP1 in the presence of ATP were identified: (i) a high-molecular-weight (HMW) band composed of Cdc48a, RecJ3, RecJ4, and aRNase J and (ii) a low-molecular-weight (LMW) band of aRNase J (Fig. 2B; Data set S1A). These proteins were not detected when the beads were coated with bovine serum albumin (BSA) or when ATP was omitted from the binding buffer. Genomic DNA did not appear to contribute to the binding, as DNase was included in the assay buffer. The ATP dependence of binding implicated Cdc48a, as Cdc48-type AAA ATPases are associated with Ub/Ubl pathways (55) and do so through ATP-dependent conformational shifts (56).

### Phenotypic analysis of mutants associated with Ubl interactome

**Fig 3:**
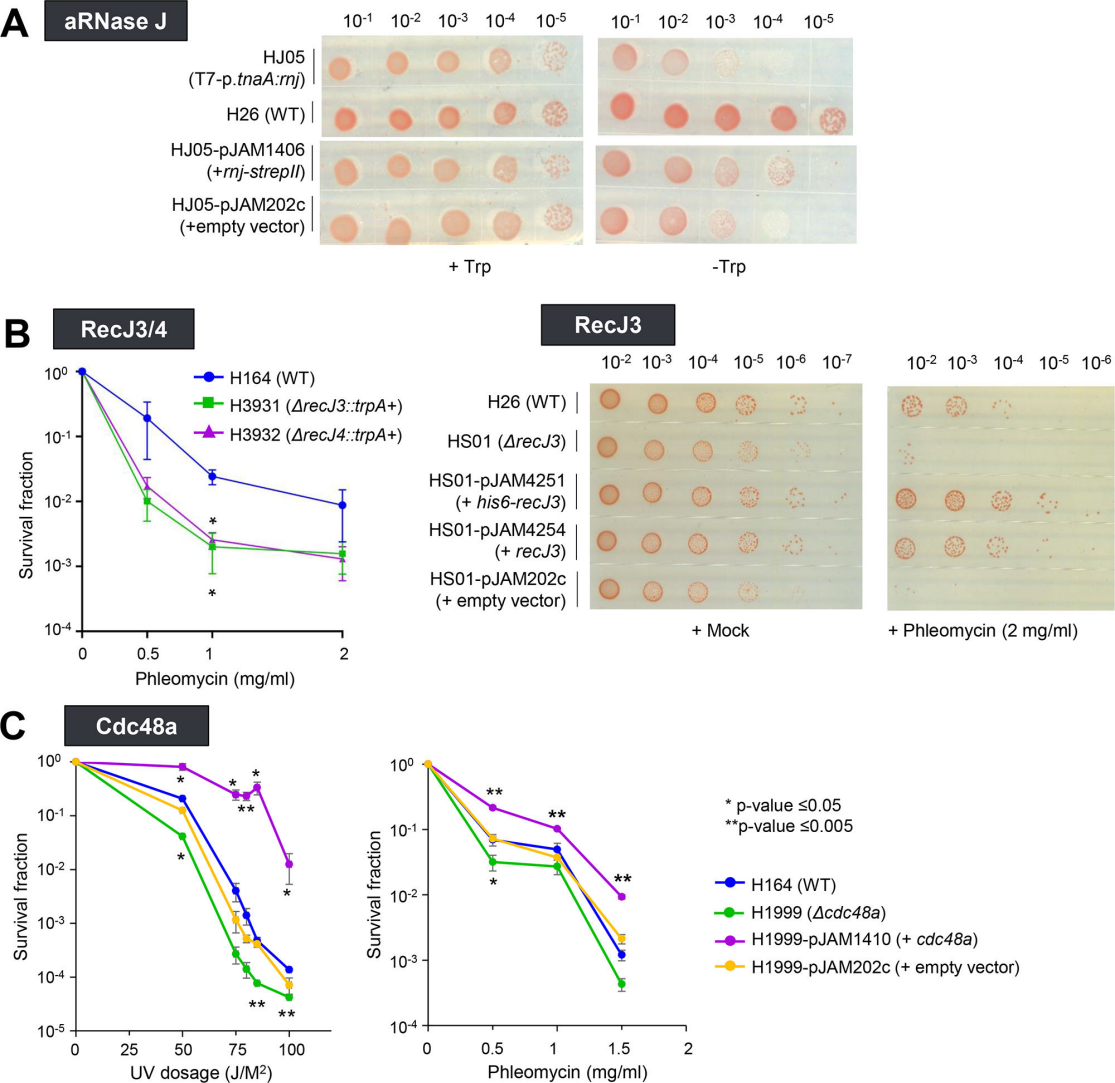
Phenotypic analysis of mutants of the Ubl interactome identified in *H. volcanii*. (**A**) aRNase J gene (*rnj*) appears essential for growth. Conditional depletion of *rnj* expression by removal of tryptophan from HJ05 (T7-p.*tnaA:rnj*) alone or with the empty vector compared to complementation by ectopic expression of *rnj-strepII*. (**B**) *recJ3* and *recJ4* genes are important for recovery after treatment with phleomycin, a DSB agent. Hypersensitivity of *ΔrecJ3* and *ΔrecJ4* mutants to phleomycin (left). Complementation of *ΔrecJ3* by ectopic expression of *his6-recJ3* and *recJ3* (right). (**C**) Cdc48a impacts recovery after treatment with UV (left) and phleomycin (right). +*cdc48a,* gene ectopically expressed from strong, constitutive (*p.rrn2*) promoter on plasmid pJAM1410; *Δcdc48a*, mutant strain. Panels B and C, **P-value <0.005 and *P-value <0.05 based on unpaired two-tailed *t*-test compared to parent strain. SEM (error bars) of at least *n* = 3 independent experiments. Images represent at least *n* = 3 independent experiments. See the Materials and Methods for details. WT, “wild-type” parent strain.

To assess the biological role of the Ubl interactome (RecJ3, RecJ4, aRNase J, and Cdc48a), the encoding genes were targeted for deletion, and the resulting mutants were analyzed for viability and recovery from DNA-damaging agents. aRNase J was suggested to be essential, as the encoding gene (*rnj*) could only be deleted from the genome when *rnj* was present on a plasmid. Additionally, the *rnj* + plasmid could not be cured from the *Δrnj* mutant (Fig. S1). To provide further evidence for the importance of *rnj*, a T7 terminator and tryptophan inducible promoter were integrated into the genome that allowed for the conditional expression of *rnj* in the presence (vs absence) of tryptophan. When compared to the parent, the resulting T7-p.*tnaA:rnj* strain was found to display wild-type growth in the presence of tryptophan and to have a pronounced reduction of growth in the absence of this amino acid (Fig. 3A). Growth of the p.*tnaA:rnj* strain under the tryptophan-depleted condition could be partially restored by ectopic expression of *rnj-strepII* (aRNase J-StrepII) but not by the empty vector control (Fig. 3A). Thus, through multiple genetic approaches, aRNase J was found to be essential in *H. volcanii*. By contrast, the *recJ3/4* genes were readily deleted (individually and together) from the *H. volcanii* genome and, thus, were not essential (Table S1). Compared to the parent, the *ΔrecJ3* and *ΔrecJ4* mutants were reduced in survival after treatment with phleomycin, a DNA-damaging agent that introduces DSBs (Fig. 3B, left). The phleomycin concentration (0.5–2 mg/mL) was 1,000-fold greater than those used to examine bacterial mutants of DNA repair, such as *E. coli* (57), and may reflect the ability of haloarchaea to thrive in extreme environments that require robust DNA repair mechanisms (58). Ectopic expression of *recJ3+* and *his6-recJ3*+ in the *ΔrecJ3* mutant restored cellular recovery from phleomycin to levels compared to (if not more robust than) the parent (Fig. 3B, right). Deletion of *cdc48a* was found to reduce the survival of cells exposed to phleomycin and ultraviolet (UV) radiation (Fig. 3C), with UV sensitivity similar if not more pronounced when compared to the *H. volcanii* DNA repair mutants *Δcas1* and *Δfen1* (59). Even more striking was the enhanced survival of cells from DNA-damaging agents when Cdc48a was expressed from a constitutive (*p2.rrn*) compared to native promoter (Fig. 3C). Overall, aRNase J was found essential for growth, while Cdc48a, RecJ3, and RecJ4 were shown to be important for the recovery of cells from DNA-damaging agents.

### RecJ3, RecJ4, and aRNase J form a complex

**Fig 4:**
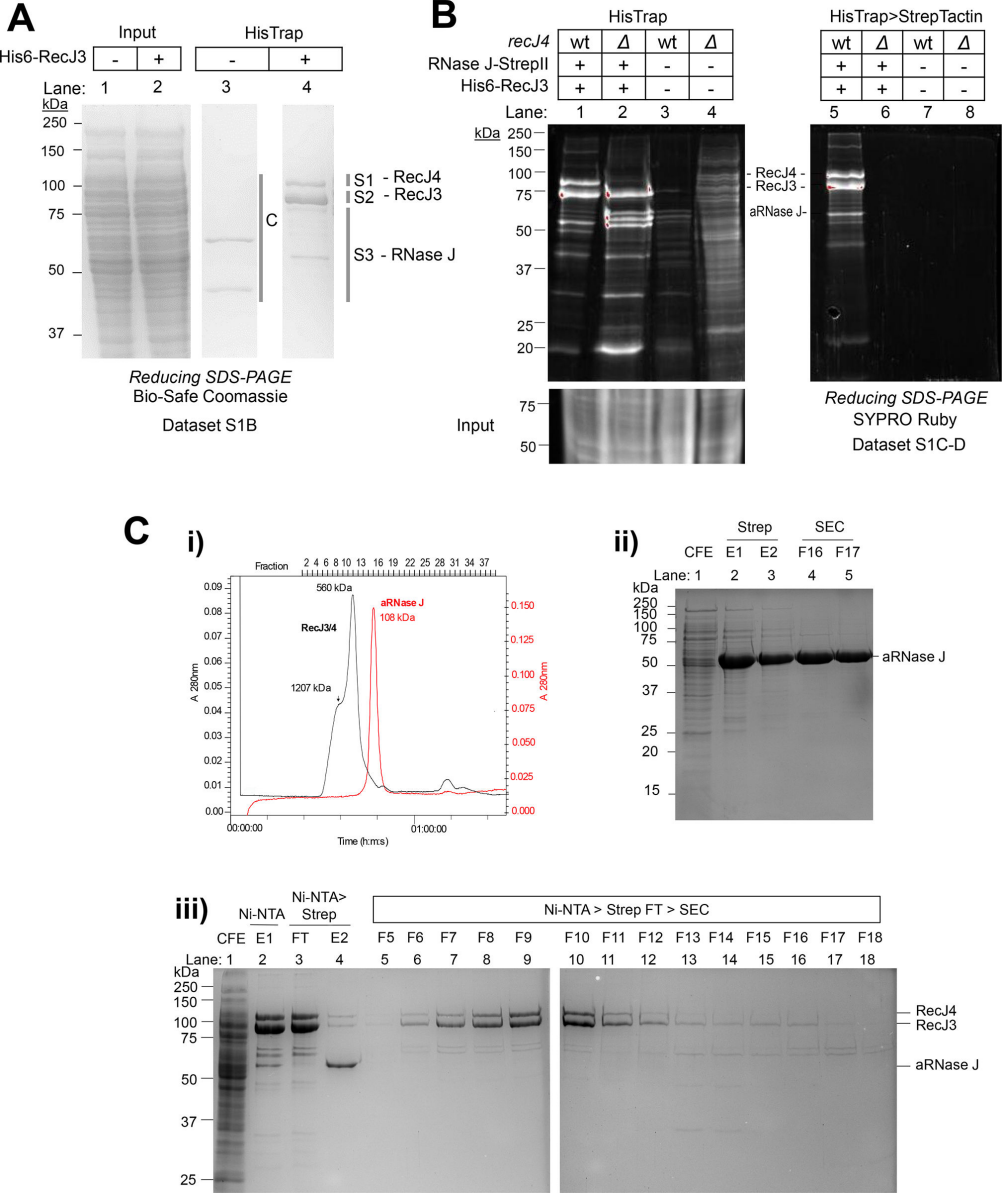
RecJ3/4-aRNase J, RecJ3/4, and aRNase J complexes purified from *H. volcanii*. (**A**) RecJ3/4-aRNase J complex purified by His-Trap. Complex was purified by His-Trap from *H. volcanii* HJ02-pJAM4251 expressing His6-RecJ3 (lanes 2, 4) compared to empty vector pJAM202c (lanes 1,3). Input (cell lysate, lanes 1–2). Vertical bars indicate regions of gel excised and analyzed by LC-MS/MS (Data set S1B). Reducing SDS-PAGE gel stained with Bio-Safe Coomassie (Bio-Rad, Hercules, CA, USA). (**B**) RecJ3/4-aRNase J complex TAP. Complex was purified from *H. volcanii* HJ02-pJAM4252 expressing His6-RecJ3 and aRNase J-StrepII (lanes 1, 5). The complex was not purified by TAP from the same strain carrying the empty vector pJAM202c (lanes 3, 7) or from *H. volcanii* HJ04-pJAM4252, an *ΔrecJ4* mutant expressing His6-RecJ3 and aRNase J-StrepII (lanes 2, 6) or its empty vector control pJAM202c (lanes 4, 8). TAP consisted of HisTrap (lanes 1–4) followed by Strep-Tactin (lanes 5–8). Input (cell lysate, bottom left panel). Left, molecular mass standards. Reducing SDS-PAGE gel stained with SYPRO Ruby. Liquid fractions of samples associated with lanes 5 and 7 were analyzed by LC-MS/MS and AQUA-based MS/MS (Data sets S1C-D). (**C**) Purification of the RecJ3/4 subcomplex and aRNase J homodimer. (i) Chromatograms of RecJ3/4 and aRNase J subcomplexes (black and red, respectively). Observed *M*_*r*_ (indicated) based on size exclusion chromatography (SEC) using a Superdex 200 Increase 10/300 Gl chromatography calibrated with molecular mass standards. (ii) aRNase J complex purified from *H. volcanii* HS01-pJAM1406 (*ΔrecJ3* expressing aRNase J-StrepII). Reducing 10% SDS-PAGE gel of cell-free extract (CFE, lane 1), Strep-Tactin fractions (E1-2, lanes 2–3), and SEC fractions (F16-17, lanes 4–5). (iii) RecJ3/4 and RecJ3/4-aRNase J complexes purified from *H. volcanii* HJ07 (*ΔrecJ3 pitA_Nph_ Δrnj* expressing His-RecJ3 and RNaseJ-StrepII). Reducing 10% SDS-PAGE gel of CFE (lane 1), Ni-NTA fractions (E1, lane 2), Ni-NTA fractions unbound (FT, lane 3) and bound to Strep-Tactin (E2, lane 4), and SEC fractions of lane 3 FT (SEC F5-18, lanes 5–18).

To further investigate the Ubl interactome, an *H. volcanii* strain (HJ02-pJAM4251) was constructed that allowed for the purification of RecJ3 with an N-terminal His6-tag (His6-). Proteins uniquely bound to His6-RecJ3 were identified by comparison to an empty vector control (HJ02-pJAM202c). The predominant proteins, excised from reducing SDS-PAGE gels and analyzed by LC-MS/MS, were found to correspond to RecJ3, RecJ4, and aRNase J (Fig. 4A; Data set S1B). To probe further into these protein-protein interactions, His6-RecJ3 and aRNase J-StrepII were expressed in *H. volcanii* (HJ02-pJAM4252) and isolated by tandem affinity purification (TAP) consisting of His-Trap and Strep-Tactin chromatography. The TAP fractions were directly analyzed by LC-MS/MS and found to be composed of three prominent proteins: RecJ3, RecJ4, and aRNase J (Fig. 4B, lane 5 vs 6; Data set S1C). AQUA-based MS/MS analysis estimated the RecJ3:RecJ4:aRNase J subunits to be in 2:2:1 stoichiometry (Data set S1D). “Low-abundant” proteins were also identified in these TAP fractions (vs empty vector) including homologs of DNA repair (UvrA and RadA), CRISPR-associated protein Cas7 (60), DnaK chaperone, and the β-CASP ribonuclease family homolog aCPSF1 (archaeal cleavage and polyadenylation specificity factor 1) (61). Overall, RecJ3, RecJ4, and aRNase J were found to be associated through protein-protein interactions by multiple strategies of purification.

### RecJ4 as an apparent scaffold of the RecJ3/4-aRNase J complex

RecJ4 appears non-catalytic based on the absence of conserved active site residues and may instead promote protein-protein interactions, as it has a large intrinsically disordered region (H104 to L236) predicted by AlphaFold (62). To examine whether RecJ4 is required for complex formation, His6-RecJ3 and aRNase J-StrepII were expressed in a *ΔrecJ4* mutant and subjected to TAP. After purification by HisTrap, the RecJ4 band of ~90 kDa was notably absent from the His-RecJ3 fractions of the *ΔrecJ4* mutant compared to the parent (Fig. 4B, lane 2 vs 1). Further purification by Strep-Tactin revealed aRNase J-StrepII to be absent from the *ΔrecJ4* fractions compared to the parent (Fig. 4B, lane 6 vs 5). These results provide evidence that RecJ4 is important for the association of RecJ3 with aRNase J.

### aRNase J and RecJ3/4 subcomplexes

To further probe the subunits of the complex, multiple strategies were used to separately purify aRNase J, RecJ3, and RecJ4. Initially, these proteins were individually expressed and purified from recombinant *E. coli* (Fig. S2A) but were found inactive in hydrolyzing RNA and/or DNA even after dialysis into “high-salt” (e.g., 2 M NaCl) buffers. Haloarchaea use a “salt-in” strategy to maintain homeostasis in hypersaline conditions, and, thus, their proteins often require synthesis, purification, and storage in “high-salt” buffers for activity and stability (63 - 65). To overcome this hurdle, aRNase J was expressed with a C-terminal StrepII tag in an *H. volcanii ΔrecJ3* mutant and purified to homogeneity by Strep-Tactin and size exclusion chromatography (SEC) (Fig. 4Ci and Cii). aRNase J purified in this manner was found to be associated as a 108-kDa homodimer. To purify the RecJ3/4 subcomplex and ensure RNase J was not present in the preparation, the following strategy was used. The plasmid expressing His-RecJ3 and aRNase J-StrepII was transformed into the *recJ3* mutant HJ02, and the *rnj* gene-encoding aRNase J (which is essential) was subsequently deleted from the genome. The resulting strain (HJ07) was used to ensure separate purification of His-RecJ3 from RNaseJ-StrepII by collecting proteins that bound the His-Trap column but flowed through the Strep-Tactin resin (Fig. 4Ciii, lane 3). These His-RecJ3 containing fractions were further purified by SEC and found to elute primarily as a 560-kDa subcomplex of RecJ3 and RecJ4 (Fig. 4Ci) in an equimolar ratio based on reducing SDS-PAGE analysis (Fig. 4Ciii, lanes 9-10). These results suggested RecJ3/4 form a tetramer of heterodimers (4 × 149 kDa), as the theoretical molecular masses of RecJ3 and RecJ4 are 70 and 79 kDa, respectively. Overall, these results reveal aRNase J to be a homodimer in the absence of RecJ3 and show RecJ3/4 to be associated in a large subcomplex of equimolar subunit ratio in the absence of aRNase J.

### Nuclease activity of RecJ3/4-RNase, aRNase J, and RecJ3/4 complexes

**Fig 5:**
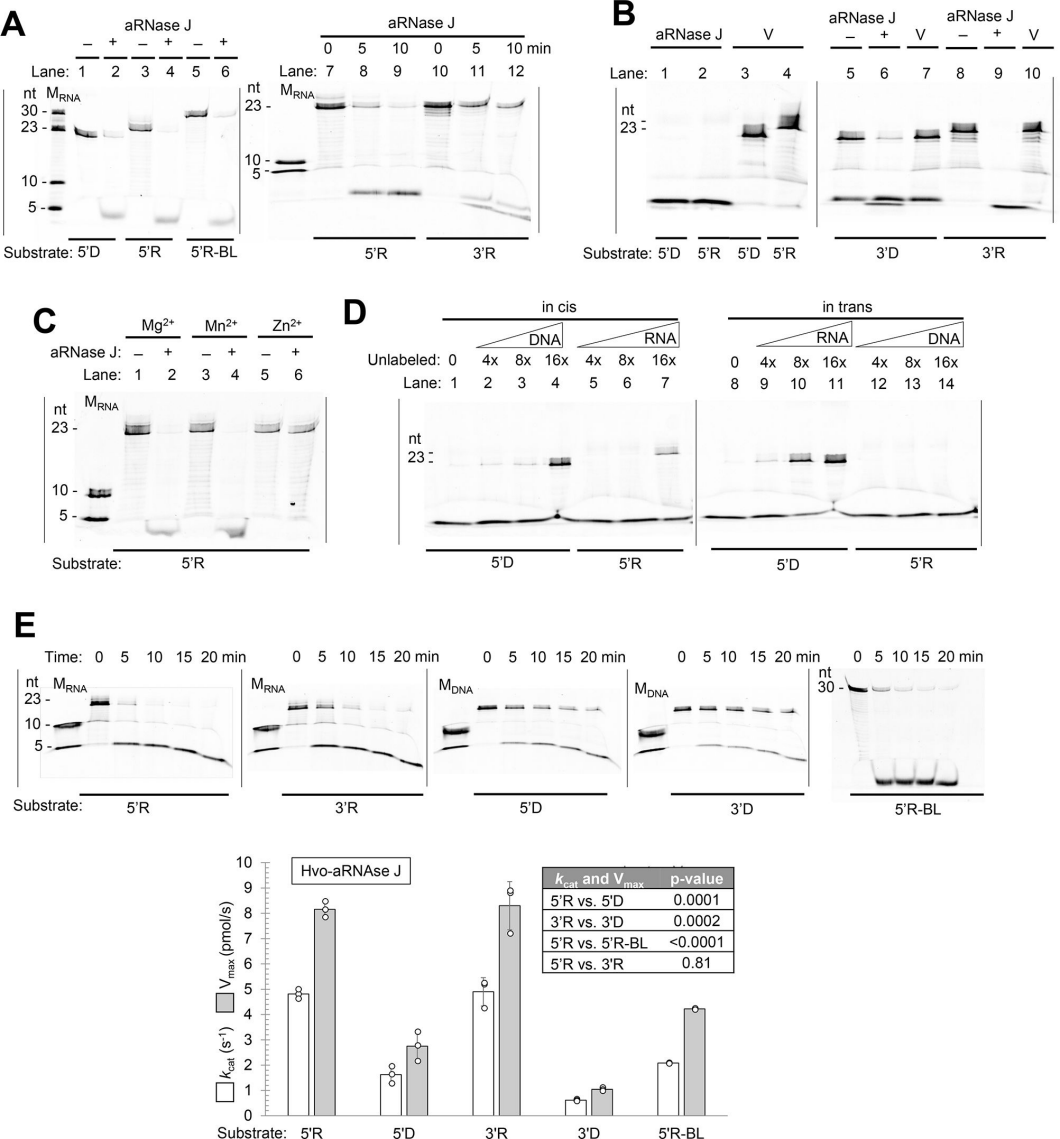
Nuclease activity of aRNase J homodimer. (**A**) aRNase J hydrolysis of RNA and ssDNA oligonucleotides. Reactions (10 µL): 50 mM HEPES (pH 7.5) with 2 M NaCl, 5 mM MnCl_2_, 5 mM MgCl_2_, 1 mM dithiothreitol (DTT), 10 nmol substrate, and aRNase J (0.5 µg, 5 pmol) (+) or mock control (−) as indicated. Reactions incubated at 50°C for 80 min, unless otherwise indicated. (**B**) aRNase J nuclease activities compared to empty vector control. Reactions as in panel A with aRNase J-StrepII homodimer purified from *H. volcanii* HS01-pJAM1406 compared to protein fractions similarly purified from *H. volcanii* HS01-pJAM202c (empty vector control, V). (**C**) aRNase J requires Mg^2+^ or Mn^2+^ (not Zn^2+^) for activity. Reactions (10 µL): aRNaseJ (8.5 pmol) or mock control (−), 3 nmol substrate, 2 M NaCl, 50 mM HEPES (pH 7.5), 1 mM DTT, and 5 mM divalent metal (MnCl_2_, ZnCl_2_, or MgCl_2_) were incubated at 50°C for 20 min (as indicated). (**D**) Unlabeled oligonucleotides impact aRNase J nuclease activity. Unlabeled DNA (in *cis*, lanes 1–4) and RNA (in *trans*, lanes 9–12) reduce aRNase J hydrolysis of 5′D, while unlabeled RNA (in *cis*, lanes 5–8) but not DNA (in *trans*, lanes 13–16) reduces aRNase J hydrolysis of 5′R. Assay conditions as in panel A with 5 nmol substrate and unlabeled DNA and RNA at the 4-, 8-, and 16-fold concentrations indicated. (**E**) aRNase J enzyme kinetics. Reactions (10 µL): aRNase J (0.17 µg, 1.7 pmol), 50 mM HEPES, pH 7.5, 2 M NaCl, 1 mM DTT, 5 mM MnCl_2_, 5 mM MgCl_2_, and 3 nmol substrate. Reactions were incubated at 50°C for 0, 5, 10, 15, and 20 min (as indicated). No hydrolysis was observed in enzyme minus reactions similarly incubated. ImageJ was used to quantify the rate of substrate hydrolysis and calculate *k*_cat_ and *V*_max_ values based on experimental triplicate. Results are experimentally reproducible. Substrates: 5′D and 3′D, TTCGGCGACTGATGTTGATTGGC (23 ntd) labeled at the 5′- and 3′-end with 6-FAM (carboxyfluorescein); 5′R and 3′R, UUCGGCGACUGAUGUUGAUUGGC (23 ntd) labeled at the 5′- and 3′-end with 6-FAM; 5′R-BL, CGAACUGCCUGGAAUCC*U*G*U*CGAACUGUAG labeled at the 5′-end with 6-FAM and internally labeled (BL, body labeled) with phosphorothioate (*). Molecular mass standards: M_RNA_, 5′ 6FAM-UUCGG and 5′ 6FAM-UUCGGCGACU with or without substrate; M_DNA_, 5′ 6FAM-TTCGG and 5′ 6FAM-TTCGGCGACT. Unlabeled DNA and RNA: TTCGGCGACTGATGTTGATTGGC and UUCGGCGACUGAUGUUGAUUGGC.

The aRNase J, RecJ3/4, and RecJ3/4-aRNase J complexes purified from *H. volcanii* were next examined for nuclease activity. Oligonucleotide substrates of ssDNA and RNA (23 nucleotides, nts) labeled at the 3′- and 5′-ends with 6-FAM (5′D, 3′D, 5′R, 3′R, see Fig. 5 legend) were used, in part, to distinguish 5′−3′ and 3′−5′ exonuclease activities, as this label should block exonuclease activity (66). The 3′ 6-FAM-labeled oligonucleotides have 5′ hydroxyl groups. Thus, the 3′R mimics RNA cleaved by metal-independent RNases (e.g., EC 4.6.1.18) and not nascent mRNA which commonly has a 5′ triphosphate group (67). An RNA substrate of 30 nts, labeled at the 5′-end with 6-FAM and including phosphorothioate modification in the body (BL, body label), was also included to examine endonuclease activity. This latter substrate (5′R-BL, see Fig. 5 legend) was designed to block 5′ exoribonuclease activity and to distinguish between enzymes that have only 3′−5′ exonuclease activity vs those that have endonuclease activity. A product of 21 nts should accumulate if the enzyme has 3′−5′ exonuclease activity and lacks endonuclease function. Alternatively, the formation of products <21 nts would indicate the enzyme has endonuclease activity.

In the homodimeric configuration, aRNase J was found to be an endonuclease that hydrolyzed all RNA and ssDNA substrates examined (Fig. 5). The hydrolyzed products were found to be less than 5 nts (Fig. 5A). The nuclease activities could be attributed to aRNase J, as proteins similarly purified from the empty vector control were not active (Fig. 5B). aRNase J was found active when Mg^2+^ or Mn^2+^ (vs Zn^2+^) was included in the assay (Fig. 5C). Hydrolysis of the 5′-end-labeled ssDNA by aRNase J was reduced when unlabeled ssDNA or RNA was added in excess to the reaction (Fig. 5D, lanes 2–4, 9–11). By comparison, aRNase J-mediated hydrolysis of the 5′end-labeled RNA (5′R) was only diminished by the addition of unlabeled RNA and not ssDNA (Fig. 5D, lanes 5–7, 12–14). These results suggested aRNase J preferred RNA over ssDNA. To further probe these findings, kinetic values were determined for aRNase J. Reaction velocities (*k*_cat_ and *V*_max_) were found to be from highest to lowest according to substrate as 5′R and 3′R > 5′R-BL > 5′D > 3′D (Fig. 5E), revealing RNA to be a preferred substrate over ssDNA. Overall, aRNase J, when purified independent of RecJ3/4, was found to be a homodimeric Mn^2+^/Mg^2+^-dependent nuclease that harbored endonuclease activity with preference for RNA over ssDNA as a substrate. Thus, aRNase J was not restricted to 5′−3′ exoribonuclease activity.

**Fig 6:**
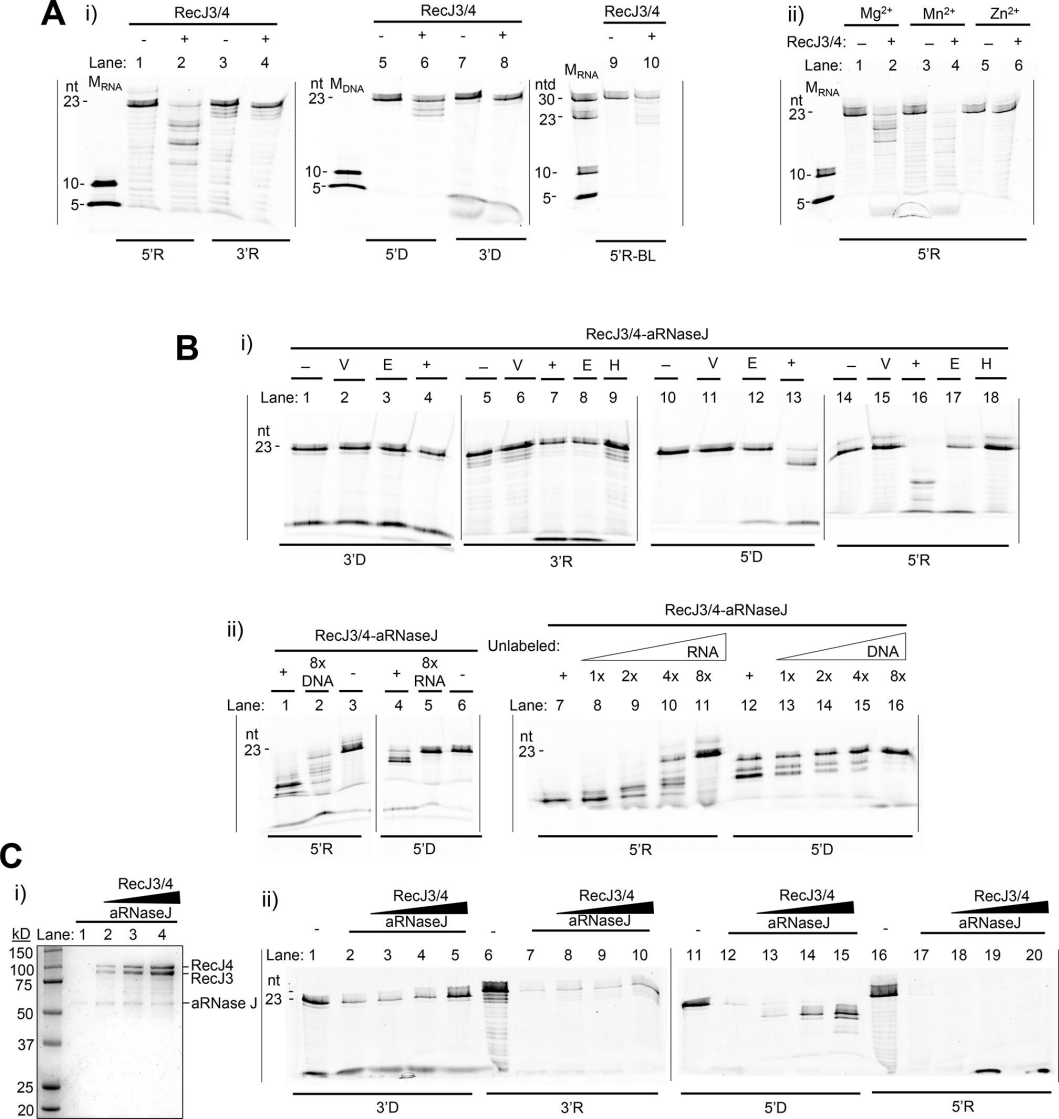
Nuclease activities of RecJ3/4 (**A**) and RecJ3/4-aRNase J (**B**) complexes and the impact of RecJ3/4 on aRNase J activity (**C**). (**A**) RecJ3/4 nuclease activities. (i) RecJ3/4 (+) activity compared to mock control (−). Reactions (10 µL): RecJ3/4 (6.7 pmol, 3.75 µg) or mock control, 2 M NaCl, 50 mM HEPES, pH 7.5, 1 mM DTT, 5 mM MnCl_2_, 5 mM MgCl_2_, and 3 nmol substrate incubated at 50°C for 80 min. (ii) Metal dependence of RecJ3/4. Standard reaction was modified to selectively include the divalent cations MnCl_2_, MgCl_2_, and ZnCl_2_ at 5 mM as indicated. (**B**) RecJ3/4-aRNase J nuclease activities. (i) RecJ3/4-aRNase J activity (+) compared to mock control (−), empty vector (V), heat treatment (H), and EDTA treatment (E) as indicated. Standard reaction (10 µL): RecJ3/4-aRNase J (0.5 µg) or controls, 20 mM Tris-HCl (pH 7.5), 2 M NaCl, 5 mM MnCl_2_, 5 mM MgCl_2_, 1 mM DTT, and 5–10 nmol substrate. Reactions were incubated at 50°C for 80 min. RecJ3/4-RNaseJ, purified at 2:2:1 stoichometry by TAP from *H. volcanii* HJ02-pJAM4252. Vector, protein fractions purified by TAP from *H. volcanii* HJ02-pJAM202c. H, RecJ3/4-aRNase J incubated at 99°C for 20 min prior to assay. E, EDTA (10 mM) included in assay. (ii) Impact of excess unlabeled oligonucleotides on RecJ3/4-aRNase J activity. Unlabeled DNA or RNA included in assay at one- to eightfold (1×, 2×, 4×, and 8×) concentration of substrate (5 nmol) as indicated. RecJ3/4-RNaseJ with no added unlabeled nucleotides (+) and TPEN [10 mM tetrakis-(2-pyridylmethyl)ethylenediamine] inhibited (−) included for comparision. (**C**) Impact of RecJ3/4 on aRNase J activity. (i) SDS-PAGE gel of aRNase J (0.5 µg per lane) mixed with increasing concentrations of RecJ3/4 (0, 1, 2, and 4 µg, lanes 1–4) and (ii) corresponding nuclease activity assays with reactions (10 µL): 20 mM Tris-HCl (pH 7.5), 2 M NaCl, 5 mM MnCl_2_, 5 mM MgCl_2_, 1 mM DTT, 5–10 nmol substrate, and enzyme as in the SDS-PAGE gel. All results were found experimentally reproducible. Molecular standards, substrates, and unlabeled oligonucleotides abbreviated as in Fig. 5.

The RecJ3/4 subcomplex was next examined for nuclease activity. RecJ3/4 was found to be most active as a 3′−5′ exonuclease with an apparent preference for RNA over ssDNA (Fig. 6Ai, lane 2 vs 6). The primary products, detected for the RecJ3/4-mediated hydrolysis of RNA and ssDNA in the 3′−5′ direction, were >10 nt. The RecJ3/4 subcomplex was found to have limited if any 5′−3′ exonuclease activity against RNA or ssDNA (Fig. 6Ai, lanes 4 and 8) and to have no RNA endonuclease activity based on finding RNA products ≥21 nts accumulated when assayed with the 5′R-BL substrate (Fig. 6Ai, lane 10). Further analysis for metal dependence revealed the RecJ3/4 complex was functional in the presence of Mg^2+^ or Mn^2+^ but not Zn^2+^ (Fig. 6Aii). Thus, when compared to aRNase J, the RecJ3/4 subcomplex had a similar divalent metal requirement of Mg^2+^ or Mn^2+^ (vs Zn^2+^); however, RecJ3/4 had limited, if any, 5′−3′ exo- or endonuclease activity and instead hydrolyzed RNA and ssDNA by a 3′−5′ exonuclease mechanism that appeared non-processive.

The nuclease activity of the RecJ3/4-aRNase J complex with 2:2:1 subunit stoichiometry was also analyzed. The complex was found to display activities that mirrored the RecJ3/4 subcomplex (Fig. 6Bi). RecJ3/4-aRNase J preferentially hydrolyzed 5′-end-labeled RNA and ssDNA oligonucleotides with limited, if any, hydrolysis observed for the 3′-end-labeled RNA or ssDNA substrates (Fig. 6Bi). These activities were specific to RecJ3/4-aRNase J when compared to protein fractions similarly purified from the empty vector control strain and were sensitive to heat and EDTA treatments (Fig. 6Bi). Further examination revealed RecJ3/4-aRNase J hydrolysis of the 5′-end-labeled ssDNA substrate (5′D) was reduced when examined in the presence of excess unlabeled RNA or ssDNA (Fig. 6Bii, lanes 5 and 16). Likewise, RecJ3/4-aRNase J hydrolysis of the 5′-end-labeled RNA substrate (5′R) was substantially reduced in the presence of excess unlabeled RNA (Fig. 6Bii, lane 11); however, the hydrolysis of 5′R appeared simulated by excess unlabeled ssDNA (Fig. 6Bii, lane 2 vs 1). These results suggest RecJ3/4-aRNase J functions primarily as a 3′−5′ exonuclease and prefers RNA over ssDNA as a substrate (with ssDNA potentially stimulating the RNase activity).

### RecJ3/4 impact on aRNase J nuclease activity

The nuclease activity profiles of the complex and subcomplexes suggested that the RecJ3/4 may influence aRNase J activities. To further investigate this possibility, increasing amounts of RecJ3/4 were added to the aRNase J homodimer and then assayed for nuclease activity (Fig. 6Ci). As the ratio of RecJ3/4 to aRNase J was increased, hydrolysis of the 3′-end-labeled ssDNA and RNA substrates (3′D and 3′R) by endonuclease and/or 5′−3′ exonuclease activities was found to be reduced (Fig. 6Cii). Hydrolysis of the 5′-end-labeled ssDNA (5′D) also appeared to shift to the formation of larger nt products (Fig. 6Cii). By contrast, hydrolysis of the 5′-end-labeled RNA (5′R) by 3′−5′ exonuclease activity was found to be robust even at high ratios of RecJ3/4 to aRNase J (Fig. 6Cii). These results suggest that RecJ3/4 influences aRNase J activity and results in a complex that functions primarily as a 3′−5′ exoribonuclease, with the non-processive 3′−5′ hydrolysis of ssDNA also observed.

### Cdc48a is loosely associated with the RecJ3/4-aRNase J complex

The Ubl interactome was further probed by analyzing various affinity-purified protein fractions by MS/MS. In brief, aRNase J-StrepII was found to co-purify with RecJ3/4 and Cdc48a (Fig. S2B; Data set S1F). RecJ4-StrepII was detected in association with RecJ3, aRNase J, Cdc48a, and Cas7 (Fig. S2B; Data set S1G). His6-Cdc48a was identified to bind a wide variety of proteins including those of translation and PTM pathways (Fig. S2B; Data set S1H) consistent with other AAA ATPases that notoriously interact with diverse protein partners and substrates (68, 69). Overall, these pull-down results provided additional evidence that RecJ3, RecJ4, and aRNase J form a complex that may transiently associate with Ubl tags through Cdc48a.

### RecJ4 and Cdc48a are Ubl modified

Diglycine remnants (+114 Da) were detected on the lysine residues of tryptic peptides derived from RecJ4-StrepII and His6-Cdc48a. The mass spectra were supported by the y- and b-ion series for each peptide (Data set S1I). These remnants were mapped to Cdc48a K210, K216, and K723 and RecJ4 K358. The *H. volcanii* strains used for protein purification encoded Ubl SAMP2, which has a -KGG C-terminal tail that would generate these footprints. Thus, Cdc48a and RecJ4 appeared samp2ylated (conjugated to SAMP2). The sites were not fully occupied by the Ubl modifier suggesting regulation, and the lysine residues were found conserved among species (Data set S1J and K). To provide further evidence for these PTMs, cell lysate was separated by reducing SDS-PAGE and analyzed by immunoblotting using antibodies raised against the related Cdc48 VCP. Cdc48a was detected as two distinct bands in *H. volcanii* “wild-type” cells and was not detected in the *Δcdc48a* mutant (Fig. S3). The faster migrating band that may be the unmodified form of Cdc48a was found to be highly abundant in the *cdc48a*^+^ overexpression strain (Fig. S3). These results are consistent with Ubl modification of Cdc48a. Interestingly, other proteins of the Ubl interactome are found Ubl modified in *H. volcanii* (53, 54, 61, 70).

### Co-occurrence patterns reveal RecJ3/4-aRNase J homologs are conserved in archaea lacking an RNA exosome

**Fig 7:**
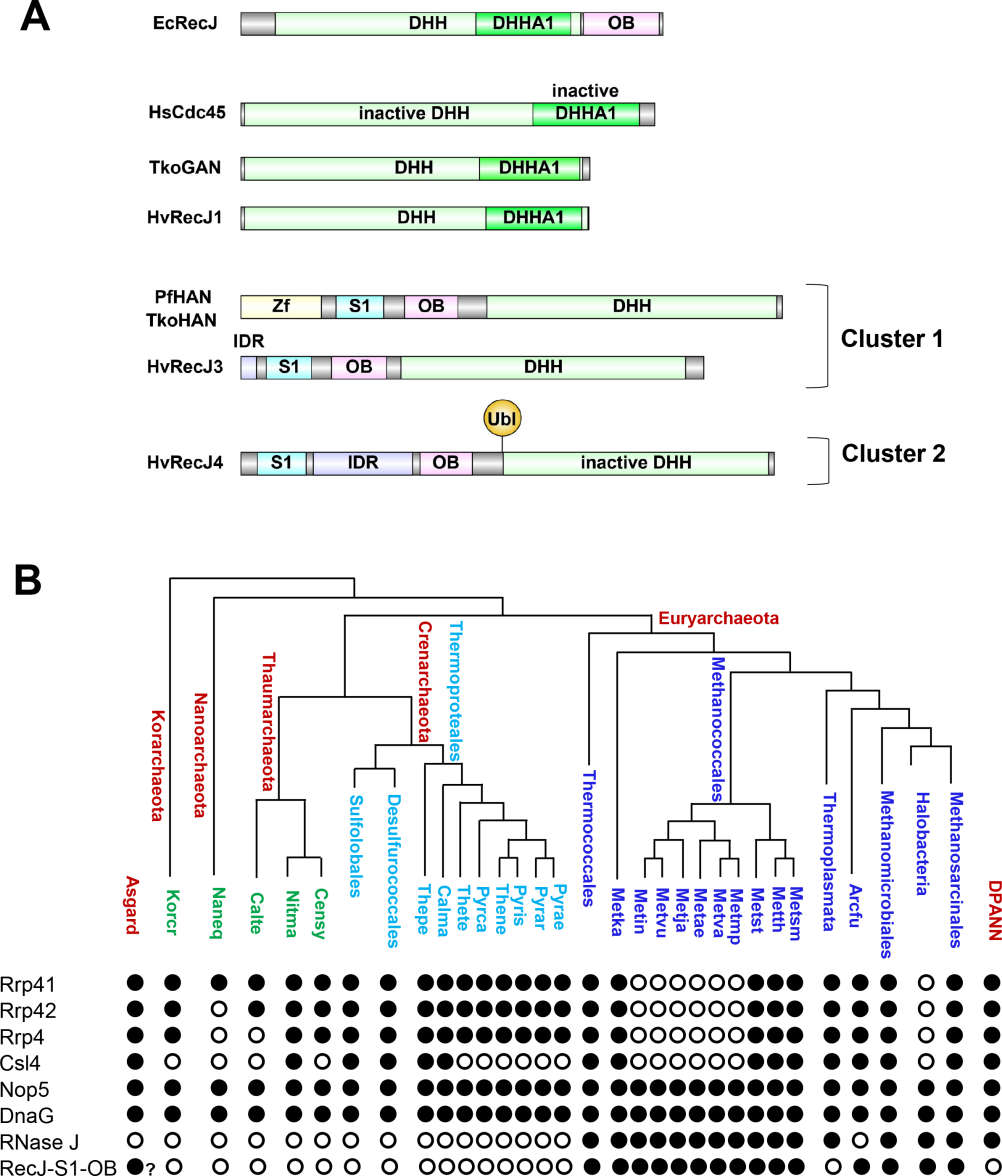
RecJ homolog domain architecture (**A**) and co-occurrence pattern of RNA exosome compared to RecJ3/4-aRNase J homologs among archaea (**B**). DHH, DHH phosphoesterase superfamily IPR038763; DHHA1, DHH associated domain 1 IPR003156; OB, nucleic acid binding OB-fold IPR012340 or RecJ OB domain IPR041122; S1, S1 RNA-binding specific domain of the OB-fold superfamily IPR022967; Zf, zinc finger motif of IPR001305. EcRecJ (P21893); *Homo sapiens* HsCdc45 (O75419); PfHAN (Q8U3Q7); TkoHAN (Q5JFJ4); and TkoGAN (Q5JGL0), with UniProt numbers in parenthesis. Clusters 1 and 2, two distinct clusters observed for RecJ homologs of IPR038763, IPR012340, and IPR022967 (met all three criteria) by sequence similarity network analysis at an alignment score of 100. Interpro (domain, family, and/or superfamily) used to determine assignments included Rrp41 (IPR011807), Rrp42 (IPR020869), Rrp4 (IPR023474), Csl4 (IPR039771), Nop5 (IPR000692), DnaG (IPR020607), RecJ/OB/S1 (IPR038763, IPR012340, and IPR022967), and RNase J (IPR004613). Arcfu, *Archaeoglobus fulgidus*; Metsm, *Methanobrevibacter smithii* ATCC 35061; Metth, *Methanothermobacter thermautotrophicus*; Metst, *Methanosphaera stadtmanae*; Metmp, *Methanococcus maripaludis* S2; Metva, *Methanococcus vannielii* SB; Metae, *Methanococcus aeolicus* Nankai-3; Metja, *Methanocaldococcus jannaschii*; Metvu, *Methanocaldococcus vulcanius* M7; Metin, *Methanocaldococcus infernus* ME; Metka, *Methanopyrus kandleri*; Pyrae, *Pyrobaculum aerophilum*; Pyrar, *Pyrobaculum arsenaticum* DSM 13514; Pyris, *Pyrobaculum islandicum* DSM 4184; Thene, *Thermoproteus neutrophilus* V24Sta; Pyrca, *Pyrobaculum calidifontis* JCM 11548; Thete, *Thermoproteus tenax*; Calma, *Caldivirga maquilingensis* IC-167; Thepe, *Thermofilum pendens* Hrk 5; Censy, *Cenarchaeum symbiosum*; Nitma, *Nitrosopumilus maritimus* SCM1; Naneq, *Nanoarchaeum equitans*; Korcr, Candidatus *Korarchaeum cryptofilum* OPF8; Calte, Candidatus *Caldiarchaeum subterraneum*. ?, few examples restricted to environmental samples. Method used to present co-occurrence data adapted from reference (35).

To understand the phylogenetic relationships of the RecJ3/4-aRNase J complex, co-occurrence patterns were compared to the archaeal RNA exosome. Taxonomic distribution patterns of ASH-Ski2, aRNase J, and Rrp41/Csl4 (RNA exosome) homologs among archaea have been analyzed by the previous study (13). Here, criteria were newly formulated to group RecJ3/4 homologs based on distinctions in protein domain architecture (Fig. 7A; Data set S2A). In this scheme, RecJ3/4 homologs were classified based on the presence of an RNA-binding S1 domain (IPR022967) and clustering to DHH phosphoesterase (IPR038763) and nucleic acid-binding OB-fold (IPR012340) superfamilies. RecJ3/4 homologs meeting these criteria were found only in certain archaea and were not present in eukaryotes, bacteria, or viruses. aRNase J classification was based on the established IPR004613 family, with phylogenetic distribution as previously described (13). By this approach, co-occurrences of RecJ3/4 and aRNase J homologs were found to be restricted to euryarchaeota and to complement archaea missing the canonical RNA exosome including all haloarchaea and certain methanogens (Fig. 7B).

### Genome synteny

As archaeal RNA exosomes are found commonly encoded in genomic neighborhoods with machinery for protein degradation, transcription, and translation (71), we next examined whether the newly identified RecJ3/4-aRNase J complex had similar associations (Fig. S4; Table S2; Data Set S2). RecJ3, RecJ4, aRNase J, and Cdc48a gene homologs were found in genomic neighborhoods with homologs of the Ubl-proteasome system, RNA degradation, DNA recombination/repair, translation, transcription, and other important cellular functions. Interestingly, RecJ3 and Cdc48 gene homologs were found in apparent operons in some methanogens, and gene homologs of Cdc48 and PRC-barrel domain proteins used in RNA processing (72) were also found in genomic synteny. Thus, while the Rec3/4-aRNase J complex differed in subunit composition from the RNA exosome, it still appeared encoded on archaeal genomes with pathways of protein degradation, transcription, translation, and other related functions.

## DISCUSSION

**Fig 8:**
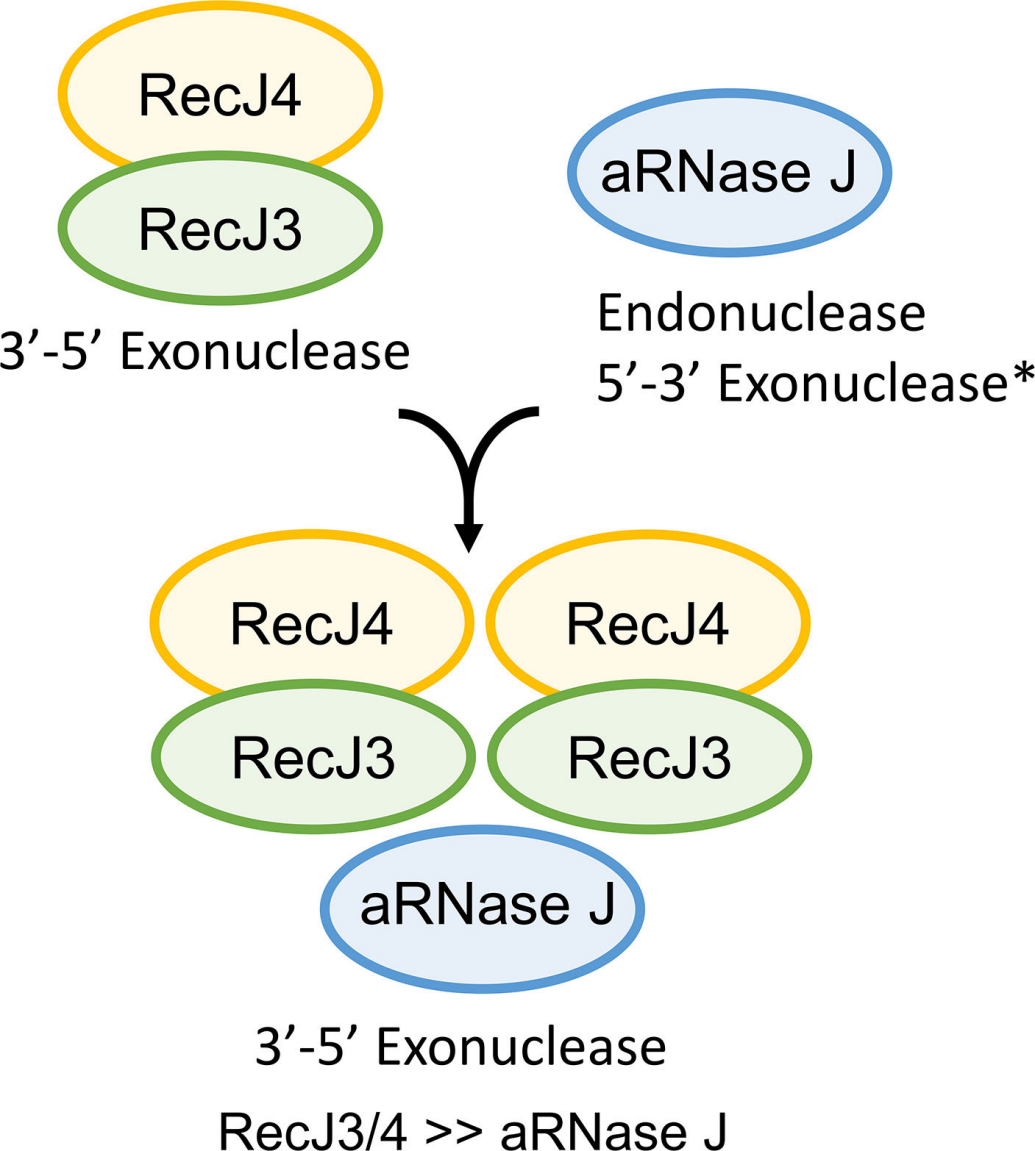
Model of *H. volcanii* RecJ3/4 and aRNase J nuclease activities in complex and subcomplex forms based on *in vitro* activity assays. RecJ3/4 >> aRNase J indicates activities observed with twofold or more abundance of RecJ3/4 to aRNase J. The RecJ3/4 subcomplex appears primarily in a tetramer of heterodimers configuration, while aRNase J alone forms a homodimer. aRNase J endoribonuclease activity was detected, while its *5′−3′ exonuclease activity is presumed based on analogy to other aRNase J enzymes.

Here, a new type of nuclease complex, composed of RecJ3, RecJ4, and aRNase J, is identified in *H. volcanii*. The complex functions primarily as a 3′−5′ exonuclease on RNA and ssDNA substrates, with the mechanism non-processive for ssDNA. Related subcomplexes could be isolated including: (i) an aRNase J homodimer that catalyzed endonuclease activity with preference for RNA over ssDNA and (ii) a 560-kDa subcomplex of RecJ3 and RecJ4 in equimolar ratio with 3′−5′ exonuclease activities paralleling that of the full RecJ3/4-aRNase J complex. Nuclease activities were found to shift primarily to 3′−5′ exonuclease when the ratio of RecJ3/4 to aRNase J was increased suggesting aRNase J activity was sequestered, altered, or outcompeted for nucleotide substrate by RecJ3/4 (Fig. 8). The nucleases were found to require Mg^2+^ or Mn^2+^ (vs Zn^2+^) and were active in high concentrations of salt (2 M NaCl), which may reflect the salt-in strategy used by *H. volcanii* to maintain homeostasis in hypersaline ecosystems. Similar salt requirements are observed for many other *H. volcanii* enzymes, e.g., proteasomes, inorganic pyrophosphatase (73, 74), but surprisingly not *H. volcanii* RNase R or RNase Z which are inactive in concentrations of >0.2 M salt (75 - 77).

The RecJ3/4-aRNase J complex was discovered based on its ATP-dependent association with Ubl SAMP1 and Cdc48a. Cdc48a is the proposed candidate that facilitates the Ubl binding of the RecJ3/4-aRNase J complex. This hypothesis is based on the relationship of Cdc48a to ATPases that are biologically linked to Ubl-/Ub-modification proteasome systems (55, 56, 68, 78 - 83). Moreover, Cdc48-type ATPases are found to alter protein-protein and/or protein-nucleic acid interactions through ATP-dependent conformational shifts (56, 82 - 84).

Cdc48a is found Ubl modified at K210, K216, and K723. These residues are conserved in diverse archaea and analogous to PTM hot spots of the related human p97 (85, 86) (Fig. S5A). Based on 3D-structural comparison (68, 87), Cdc48a K210 and K216 are residues predicted to be located at an intersubunit interface that may engage in substrate protein unraveling, while K723 appears positioned where the substrate protein, once unfolded, would pass through the ATPase pore. Thus, the observed Ubl modifications of Cdc48a are in positions that may impact biological function. Other members, of the *H. volcanii* AAA ATPase network, are also post-translationally modified (51, 88).

aRNase J is found essential in *H. volcanii*. Central roles for RNase J (IPR004613) family proteins have been observed in other domains of life. In *Bacillus subtilis*, RNase J1 mutations majorly impact cell morphology, sporulation, competence (89), and the resolution of stalled transcription complexes (24). *Staphylococcus aureus* RNase J mutations cause global defects in RNA maturation and degradation (90). In plant chloroplasts, RNase J null mutants are embryo-lethal (91). Our finding that *H. volcanii* (Hvo-) aRNase J is essential suggests this enzyme has a key role in RNA turnover and is in contrast to the *Thermococcus barophilus* Tba-aRNase J which is non-essential (13). One notable difference, which may explain these findings, is that *T. barophilus* encodes an RNA exosome that is central to RNA metabolism, whereas *H. volcanii* does not.

RecJ3, RecJ4, and Cdc48a are shown to be important for the recovery of *H. volcanii* from DNA-damaging agents. Correlating these phenotypes with the *in vitro* activity assays suggests the RecJ3/4 3′−5′ exonuclease activities and/or the constraint these proteins have on Hvo-aRNase J endonuclease activity may be associated with DNA repair. Mounting evidence reveals RNA and RNA-processing enzymes are important in DNA recombination and repair (5, 6). For example, DNA:RNA hybrids at DNA break sites facilitate homologous recombination and include the recruitment of RNases such as RNase H2 and RNA exosomes in eukaryotes (6).

RecJ3 is found tightly associated with RecJ4 in *H. volcanii*, suggesting the annotation of HVO_1018 (RecJ3) as a HAN may need to be broadened. RecJ3 and Hef are observed to be among the top 200 proteins that co-purify with Ubl SAMP3 in *H. volcanii* (54). Other proteins that co-purify in the Ubl network (54) include Hvo-aRNase J, Cdc48a, and other homologs of DNA repair, recombination, and nucleotide hydrolysis such as aCPSF1 (HVO_0874), CRISPR Cas7/8b (HVO_A0207/HVO_A0206), RadA (HVO_0104), enolase (HVO_2774), Tif5B (HVO_1963), UvrA (HVO_0393), NthB (HVO_0878), AP endonuclease (HVO_2322), and MutS/L (HVO_0552/HVO_0551). While these findings provide evidence that RecJ3 and Hef are members of a Ubl interactome (54), this level of selection from a theoretical proteome of 3,996 proteins (Uniref UP000008243) is not conclusive evidence that RecJ3 and Hef form a complex in *H. volcanii*.

RecJ4 functions as an apparent scaffold for the association of Hvo-aRNase J with RecJ3. Consistent with this role, RecJ4 lacks conserved active site residues (Fig. S5B) and captures Hvo-aRNase J and RecJ3 when used in affinity-tagged pull-down assays. Furthermore, a *recJ4* mutation disrupts the association of RecJ3 with Hvo-aRNase J. RecJ4 has an extensive IDR that spans residues 100–235 sandwiched between S1 and OB-fold domains including an acidic patch at positions 185–227 (Fig. 7A). This IDR is related in primary sequence to RecJ homologs that cluster to arCOG00429. RecJ3 also has an IDR; however, this region spans only 22 residues. As IDRs often provide binding sites for protein partners (92), this may explain why RecJ4 is required for Hvo-aRNase J to co-purify with RecJ3.

Protein partners of RNase J (IPR004613) family proteins are widespread and diverse. In bacteria, RNase J assembles into multisubunit RNA degradosomes and associates with translating ribosomes, chaperones, and membranes (93 - 98). These types of interactions appear to be promoted by N- and C-terminal extensions and/or fusions to other types of domains (e.g., FtsK, DnaK, MFS, and UppP/BacA) (Fig. S5C and 5D). Surprisingly, aRNase J proteins are missing these extensions and extra domains, yet still bind protein partners as exemplified by Hvo-aRNase J binding to RecJ3/4 (this study) as well as the association of *Pyrococcus abyssi* Pab-aRNase J with a Ski2-like helicase linked to the RNA exosome (13). Whether these interactions are regulated by PTMs remains to be determined. RNase J is covalently attached to Pup (a small, intrinsically disordered prokaryotic Ubl) in mycobacteria, while aRNase J (along with its protein partners) is found lysine acetylated in haloarchaea (99, 100).

Hvo-aRNase J catalyzes endoribonuclease activity and, thus, is not restricted to the 5′−3′ exonuclease activity typical of other aRNase J homologs. Hvo-aRNase J hydrolyzed all oligonucleotide substrates examined in an Mg^2+^/Mn^2+^ (vs Zn^2+^)-dependent manner and displayed a preference for RNA vs ssDNA based on *k*_cat_ and *V*_max_ values determined at saturating substrate (>1,500-fold molar excess). This type of profile is more typical of bacterial RNase J enzymes which can function as 5′−3′ exo- and/or endoribonucleases (7, 18 - 23).

Several possibilities may explain why Hvo-aRNase J has endonuclease activity (and is not restricted to 5′−3′ exonuclease activity). (i) Hvo-aRNase J shares only 33%–52% amino acid sequence identity with the characterized aRNase J enzymes. (ii) Hvo-aRNase J forms a homodimer, while the other aRNase J characterized at this level (i.e., Mpy-aRNase J) forms a homotetramer with a dimer-dimer interface apparently immobilizing the RNA-binding channel (15). The monomeric unit of Hvo-aRNase J is predicted to have an extreme acidic shell (Fig. S5E), which may promote this different subunit configuration. In further support of this possibility, bacterial RNase J enzymes that are endonucleases use entirely different subunit interfaces than Mpy-aRNase J to associate in dimers and tetramers (23). (iii) Hvo-aRNase J functions in 2 M NaCl with catalytic Mg^2+^ or Mn^2+^ ions, thus, contrasting with other aRNase J enzymes which use Zn^2+^. (iv) Hvo-aRNase J is purified from *H. volcanii* (vs recombinant *E. coli*) adding the possibility that PTMs alter its activity (e.g., lysine residues at positions 112, 120, 191, 263, and 278 are found acetylated) (100). (v) The 3D modeling of Hvo-aRNase J suggests conserved active site residues (e.g., Ser250, His391, and His83) are located on larger loops when compared to Mpy-aRNase J (Alpha-fold AF-D4GW49-F1 vs PDB: 6LLB) (15), which could provide greater flexibility in the nuclease mechanism. (vi) Longer or highly structured RNA substrates may be required for Hvo-aRNase J to strictly favor 5′−3′ exoribonucleolytic activity.

The RecJ3/4-aRNAse J complex may provide a hub for RNA degradation in *H. volcanii*, as this archaeon lacks an RNA exosome and polyadenylated RNA (101). Many archaea harbor aRNase J enzymes that appear to engage with RNA exosomes: RNA-degrading machines with 3′−5′ exoribonuclease and polyadenylation activities (13, 101). The polyadenylated poly(A) tails at the 3′-end of RNA can provide a toehold for 3′−5′ exonucleases to degrade the RNA (102). At one time, RNase R was hypothesized to be the sole exoribonuclease of *H. volcanii*, but this enzyme is not active in “high-salt” conditions (76, 77) that mimic the cytosol of this organism (103). Here, we find RecJ3/4 to mediate 3′–5′ exoribonuclease activity and to associate with aRNase J in high-salt conditions. These types of activities and protein-protein interactions may provide functional replacements for the aRNase J and RNA exosome associations observed in other archaea.

Overall, RecJ3/4-aRNase J is a newly identified nuclease complex. This complex appears conserved in halophilic and methanogenic archaea that do not encode an RNA exosome and is strikingly absent from the archaeal TACK group, members of which have RNA exosomes. The RecJ3/4-aRNase J complex associates with Ubl SAMP1 (likely *via* Cdc48a). This binding is speculated to localize and/or coordinate the degradation of nucleic acids in the cell, including DNA repair based on the *ΔrecJ3*, *ΔrecJ4,* and *Δcdc48a* mutant phenotypes. Binding to Hvo-RecJ3/4 reduces the endonuclease activity detected for Hvo-aRNase J and renders the overall function of the complex as a 3′−5′ exonuclease. Interestingly, ssDNA appears to stimulate the hydrolysis of the 5′-end-labeled RNA substrate by the RecJ3/4-aRNase J complex, consistent with the possibility that this nuclease complex could play a role in resolving DNA:RNA hybrids or R-loops that actively form at DNA double-strand breaks (104). Whether the RecJ3/4-bound Hvo-aRNase J is targeted for degradation by the Ubl-proteasome system remains to be determined. The Archaeal Proteomic Project (ArcPP), an assembly of proteomic data sets derived from *H. volcanii* grown in different labs and environmental conditions (105), reveals Hvo-aRNase J along with RecJ3, RecJ4, and Cdc48a that are detected in all conditions examined. By contrast, the Ubl SAMPs are identified only in a subset of the proteomic data sets. While not quantitative, these results point toward environmental cues signaling shifts in the protein abundance of Ubl SAMP and not the subunits of the nuclease complex. Thus, the Ubls (via Cdc48a) may regulate the protein-protein interactions of the RecJ3/4-aRNase J complex instead of targeting its subunits for degradation.

## MATERIALS AND METHODS

### Materials

Biochemicals were from Sigma-Aldrich (St. Louis, MO, USA) or Alfa Aesar by Thermo Fisher Scientific (Tewksbury, MA, USA). Other organic and inorganic analytical grade chemicals were from Fisher Scientific (Atlanta, GA, USA). Restriction endonucleases, T4 DNA ligase, and Phusion polymerase were from New England Biolabs (Ipswich, MA, USA). Taq and Pfu DNA polymerases were from Bioline (Taunton, MA, USA) and Stratagene (La Jolla, CA, USA), respectively. Desalted oligonucleotides were from Integrated DNA Technologies (Coralville, IA, USA) and Eurofins Genomics (Louisville, KY, USA). DNA sequencing was by Eton Bioscience Inc. (Research Triangle Park, NC, USA). Proteomics was performed at UF Interdisciplinary Center for Biotechnology Research (ICBR, Gainesville, FL, USA). Strep-Tactin Superflow Plus Resin (U.S. Cat. No. 1057978) was from Qiagen (Germantown, MD, USA). HisTrap HP 5 mL columns (GE Healthcare, Cat. No. 29-0510-215) were from Sigma-Aldrich. Cellulose acetate membranes, 0.2 µm, for protein filtration were from Thermo Scientific (Waltham, MA, USA). Phleomycin (Cat. No. ant-ph-1) was from InvivoGen (San Diego, CA, USA).

### Strains and media

Strains, plasmids, and primers used in this study are summarized in Table S1. *E. coli* GM2163 was used for the replication of plasmid DNA prior to transformation into *H. volcanii* strains according to standard methods (106). *E. coli* strains were grown at 37°C in Luria-Bertani (LB) medium (unless indicated as 25°C). *H. volcanii* strains were grown at 42°C in ATCC 974 *Halobacterium* medium, Casamino acids (CA) plus medium, Hv-YPC (yeast extract, peptone, and CA) medium, and Hv-Min medium (0.1% lactic acid, 0.09% succinic acid, and 0.01% glycerol served as carbon source) as described in the *Halohandbook* (106). Hv-Min was supplemented with 50 µg/mL uracil from a 50-mg/mL stock dissolved in DMSO (Hv-Min-Ura+). Cells were grown in liquid cultures with rotary shaking at 200 rpm and on a solid medium. Media were supplemented per liter with ampicillin (Amp, 100 mg), kanamycin (Km, 50 mg), chloramphenicol (Cm, 30 mg), novobiocin (Nv, 0.2–0.3 mg), 5-fluoroorotic acid (5-FOA, 50 mg), and/or uracil (50 mg) as needed unless otherwise indicated. Growth was monitored by measuring optical density at 600 nm (OD_600_), where 1 OD_600_ unit equals approximately 1 × 10^9^ colony-forming units (CFU)/mL. Once constructed and verified, all *H. volcanii* strains were stored at −80°C in 20% (vol/vol) glycerol stocks. To culture, the *H. volcanii* strains were streaked from −80°C onto ATCC974 plates and incubated for 4–5 days at 42°C for a single colony. Media were supplemented with Nv for *H. volcanii* strains carrying the pJAM plasmids.

### Conditional depletion and mutagenesis of *rnj* (aRNase J) in *H. volcanii*

To analyze the role of *rnj* (aRNase J) in *H. volcanii*, two approaches were employed. First, the *pyrE2*-based pop-in pop-out method (107) was used to target the *rnj* gene for deletion by homologous replacement from the H26 genome in the presence and absence of plasmids pJAM4253 (*rnj+*) and pJAM202c (empty vector). Second, the tryptophan-inducible promoter P*tnaA* was used to control the expression of *rnj* in the H26 genome. To construct the plasmid for insertion of the T_7_-P*tnaA* fragment upstream of *rnj* in the H26 genome, PCR amplified P*tnaA* from *H. volcanii* genomic DNA was fused to the 3′ end of the T7 terminator by overlap extension PCR. The resulting fragment was cloned into the *Xba*I and *Nde*I sites of pJAM1406. The 500-bp flanking fragment upstream of *rnj* was inserted into the *Ale*I and *Xba*I sites of this intermediate plasmid to generate the final plasmid pJAM4259, which was propagated in GM2163 and then used to modify the genome of H26 by *pyrE2*-based homologous recombination to obtain strain HJ05. For complementation assay, HJ05 was separately transformed with plasmids pJAM1406 (+*rnj-strepII*) and pJAM202c (the empty vector). The strains were inoculated with a single colony into 3 mL Hv-Min-Ura+ medium and grown to OD_600_ of 0.6–0.8 in a rotary shaker at 200 rpm and 42°C till the OD_600_ reached 0.6–0.8. Cells were subcultured from the starting OD_600_ of 0.02 to OD_600_ 0.6–0.8 with the same condition described above. This subculture step was repeated. The cells were then diluted to 10^−2^–10^−7^ in 18% saline water and spotted (20 µL each dilution) onto Hv-YPC plates. After spotting, the plates were air dried and incubated in the dark at 42°C for 5 d.

### Construction of *H. volcanii* expression plasmids

For the preparation of the His6-RecJ3 expression plasmid pJAM4251, the gene fragment encoding *recJ3* was amplified by PCR from *H. volcanii* genomic DNA with the primer set RecJ3F_ NdeI and RecJ3TAAr_XhoI and cloned into plasmid pJAM503. A similar approach was used to construct the aRNase J-StrepII expression plasmid pJAM1406 using primer set HVO_2724 NdeI and HVO_2724 KpnI and cloning into plasmid pJAM809. To construct the expression plasmid pJAM4252, carrying tandem expression cassette p2.*rrn: his6-recj3* following with p.*fdx: rnj-strepII*, an overlap extension PCR method was used to splice the promoter P*fdx* and *rnj-strepII* (108); the resulting expression cassette was then cloned into the *Blp*I site of pJAM4251. Here, a strong promoter, *H. volcanii* ferredoxin promoter P*fdx*, was used for controlling the expression of the *rnj* (aRNase J) gene. The sequencing-confirmed plasmids were transformed into GM2163 to propagate *dam*^−^ genotype plasmids and transformed into *H. volcanii* for protein expression. His6-RecJ3 and aRNase J-StrepII were expressed from plasmid pJAM4252 in the host strain HJ02, which was constructed by deleting *recJ3* and replacing the His-rich *H. volcanii pitA* with *Natronomonas pharonis pitA* (*pitA_Nph_*) to avoid His-rich PitA contamination during Ni^2+^ column chromatography as previously observed (109, 110). HJ07 was also used as a host strain for the expression of His-RecJ3 and aRNase J-StrepII from pJAM4252 and is a *Δrnj* derivative of HJ02. The *pitA_Nph_* replacement was also used to construct HJ04 (H26 *ΔrecJ4 pitA_Nph_*).

### Phleomycin and UV stress assays

Strains H164 (parent), H1999 (*Δcdc48a*), H1999-pJAM1410 (*+cdc48* a), and H1999-pJAM202c (empty vector) were used to examine *cdc48a* function. These strains were grown in 5 mL Hv-YPC medium to late log phase (OD_600_ of ~0.8) in 13 × 100 mm culture tubes at 42°C in an orbital shaker (200 rpm). Cells were subcultured to a starting OD_600_ of 0.02 and grown to log phase (OD_600_ of 0.4) in 5-mL Hv-YPC medium. Phleomycin (20 mg/mL stock in water) was added to final concentrations of 0, 0.5, 1.0, and 1.5 mg/mL in the 5-mL cultures for 1 h with intermittent orbital shaking. Deionized water was included as a mock control for comparison. The phleomycin-treated cells were diluted to 10^−2^–10^−7^ in 30% saline water and spotted (20 µL per dilution) onto Hv-YPC plates. After spotting, the plates were air dried and incubated in the dark at 42°C (6 d). For UV treatment, the cells grown to log phase in Hv-YPC medium were diluted in 30% saline water to 10^−1^–10^−5^ and spotted (20 µL per dilution) onto Hv-YPC plates. After spotting, the plates were air dried and exposed to UV dosages (0, 50, 75, 80, 85, and 100 J/M^2^). Immediately after UV treatment, the plates were incubated in the dark at 42°C (3 d). Strains H26 (parent), HS01 (*∆recJ3*), HS01-pJAM4251 (+*his6-recJ3*), HS01-pJAM4254 (+*recJ3*), and HS01-pJAM202c (empty vector) were used to examine *recJ3* function. These strains were inoculated from isolated colonies into 3 mL Hv-Min-Ura+ medium. Medium was supplemented with 0.3 µg/mL novobiocin for strains with plasmids. Cells were grown to late log phase (OD_600_ of 0.6–0.8). Cells were subcultured to a starting OD_600_ of 0.02 and grown to log phase (OD_600_ of 0.5–0.6) in 3 mL Hv-Min-Ura+ medium. The cells were treated in triplicate with phleomycin (2 mg/mL) in the 3-mL cultures for 1 h with intermittent orbital shaking. A mock control of equal volume of deionized water was included for comparison. After treatment, the cells were diluted to 10^−2^–10^−7^ in 18% saline water and were spotted (20 µL each dilution) onto Hv-YPC plates. After spotting, the plates were air dried and incubated in the dark at 42°C for 3 d. A similar approach was used for comparative assay H164 (parent), H3931 (*ΔrecJ3::trpA+*), and H3932 (*ΔrecJ4::trpA*+) with the following modifications. Hv-YPC medium was used throughout the assay. After 1 h treatment with phleomycin (0, 0.5, 1, and 2 mg/mL), the cells were harvested and resuspended in fresh YPC medium prior to diluting/plating. All experiments described above were performed in triplicate and included biological triplicates within each experiment.

### Protein concentration and high-salt strategy

Protein concentration was determined by Pierce BCA Protein Assay Kit (Thermo Fisher Scientific, Waltham, MA, USA) using the supplied BSA as a standard. Protein purification methods that required complex stability and/or enzyme activity were performed in buffers supplemented with 2 M salt. The high salt was included in the buffer to maintain the stability of the “salt-loving” proteins common to the haloarchaea (63, 64, 111). Bovine pancreas DNase I (Alfa Aesar, Cat. No. J62229) was included in lysis buffers that were found active in hydrolyzing *H. volcanii* genomic DNA (400 µg/mL) at conditions mimicking protein purification (i.e., DNase I at 4 µg/mL in high-salt lysis buffer and incubations on ice for 6 h and room temperature for 2 h).

### Ubl purification

*E. coli* Rosetta (DE3)-pJAM1131 was used for purification of the Ubl SAMP1 with an N-terminal Flag-His6-tag (the Ubl of this study). The strain was freshly transformed and inoculated into LB medium supplemented with Km and Cm (500 mL medium per 2.8 L Fernbach flask). Cells were cultured at 25°C (200 rpm). Isopropyl β-d-1-thiogalactopyranoside (IPTG) was added to a final concentration of 0.4 mM at log phase (OD_600_ 0.4–0.6 units) and cultivation was continued. After 8-h induction with IPTG, the cells were harvested by centrifugation (3,000 × *g* for 10 min at 4°C). The cells were washed in ice-chilled low-salt buffer (20 mM HEPES, pH 7.5, 150 mM NaCl) and stored at −80°C as cell pellets until lysis. Cells (3 g wet weight) were resuspended in 12 mL lysis buffer (20 mM HEPES, pH 7.5, 150 mM NaCl, 40 mM imidazole, 4 µg/mL DNase, 1 mM PMSF) and lysed thrice by French press (2,000 psi) (minimum high ratio of 140, Glen-Mills, NJ, USA). An equal volume of dilution buffer (20 mM HEPES, 4 M NaCl, pH 7.5) was added to the lysed cells. Cell lysate was clarified by centrifugation (9,200 × *g* twice for 15 min at 4°C) and filtration (0.2 µm, cellulose acetate membrane). Protein sample was applied to a His Trap HP column pre-equilibrated in buffer (20 mM HEPES, pH 7.5, 2 M NaCl, 40 mM imidazole) and washed with the same buffer. The Ubl was eluted using 500 mM imidazole in 20 mM HEPES, 2M NaCl, and pH 7.5. The eluate was concentrated to a final volume of 500 µL using a Vivaspin centrifugal concentrator (3 MWCO, GE Healthcare). Concentrated samples were applied to a Superdex 75 Increase 10/30o (FPLC column, Cytiva, Marlborough, MA, USA) equilibrated with HEPES high-salt buffer (20 mM HEPES, 2 M NaCl, pH 7.5) at a flow rate of 0.2 mL/min, and 0.5 mL fractions were collected. Fractions containing the Ubl were pooled and stored at 4°C.

### Ubl coupling to amine reactive beads

The Ubl was coupled to aldehyde-activated agarose beads (AminoLink Plus Resin) by reductive amination at room temperature according to supplier (Thermo Scientific, Waltham, MA, USA) with the following modifications. Immediately prior to coupling, the Ubl was dialyzed twice against high-salt PBS buffer (2 M NaCl, 0.1 M sodium phosphate, pH 7.2) at 4°C. Likewise, the AminoLink Plus Resin (2 mL) was equilibrated by adding three resin-bed volumes of high-salt PBS buffer at room temperature. The Ubl sample (5.0 and 8.5 mg protein) was added to the column resin (experimental replicates 1 and 2, respectively). Coupling was initiated by the addition of 40 µL of 5 M NaCNBH_3_ in 1 M NaOH. The mixture was rocked overnight at room temperature. After coupling, the flow through was collected to estimate the coupling efficiency of the Ubl through protein estimation. The remaining active sites were blocked by an initial wash with 4 mL quenching buffer (1 M Tris HCl, 0.05% NaN_3_, pH 7.5) and subsequent addition of 2 mL quenching buffer and 40 µL of 5 M NaCNBH_3_ in 1 M NaOH. The sample was gently rocked at room temperature for 30 min. The column was washed with 10 mL (five resin-bed volumes) of wash solution (2M NaCl, 0.05% NaN_3_) to remove the non-coupled protein and unreactive cyanoborohydride. The Ubl amino groups available for covalent linkage resided in the N-terminal region of the polypeptide, including the N-terminal α-amino group and ɛ-amino groups of the lysine residues (Flag-tag K3 and K8 and SAMP1 K4). BSA (BCA protein standard) was similarly coupled to the beads as a control.

### Cell lysate preparation for Ubl pull-down assay

*H. volcanii* NH02-pJAM957, a ∆*samp1-3* ∆*ubaA* mutant ectopically expressing UbaA-StrepII, was grown in ATCC974 medium with 100 mM DMSO to stationary phase at 42°C (2 × 1 L culture in 2.8 L Fernbach flask, 200 rpm; derived from biological triplicate inoculum). Cells were harvested by centrifugation (3,000 × *g* for 10 min at 4°C) and stored at −20°C as pellets (3 g wet weight) until use. On the day of the pull-down assay, the *H. volcanii* cell pellets were resuspended in ice-cold high-salt PBS buffer (0.1 M sodium phosphate buffer, pH 7.5, 2 M NaCl, 1 mM DTT, 1 mM PMSF, and 4 µg/mL DNase) by pipetting in and out. Cells were lysed by French press (twice at 2,000 psi), and the cell lysate was clarified by centrifugation (16,264 × *g* for 20 min at 4°C). The supernatant was transferred carefully into a fresh tube without disturbing the pelleted cell debris. The cell-free extract was concentrated by dialysis against polyethylene glycol (PEG) 8000, where the cell-free extract was packed in SnakeSkin Dialysis Tubing (3K MWCO, Thermo Scientific) and incubated at 4°C in a container filled with PEG 8000. The cell-free extract was concentrated to a final volume of 3 mL, after which it was dialyzed against high-salt PBS buffer (2 M NaCl, 0.1 M sodium phosphate, 0.2 mM DTT, pH 7.2) at 4°C. Immediately prior to pull-down assay, 5 mM ATP was added to the cell-free extract. Preparations that excluded the ATP supplementation were also analyzed for comparison.

### Ubl pull-down assay

The Ubl-coupled beads were equilibrated with three-column volumes (6 mL) of high-salt PBS buffer. The cell lysate prepared for pull-down assay at 4°C or on ice was applied to the Ubl-coupled column (3 mL total) at room temperature. The protein-bead slurry was incubated at 4°C with gentle rocking (rocker M71015, Barnstead International, Dubuque, IA, USA) for 4 h per each 2 mL application. Washes were performed at 4°C or on ice. The column was washed with 40 mL high-salt PBS buffer (the flow through was collected to determine the binding efficiency). Proteins bound to the column were eluted at room temperature by the addition of 8 mL of 0.1 M glycine-HCl buffer at pH 2.5 and collected as 1 mL fractions in tubes with 50 µL neutralization buffer (1 M Tris-HCl, pH 8.5). Elutes were stored at −20°C or −80°C (long term). Proteins were separated by SDS-PAGE and visualized by staining with SYPRO Ruby. Cell lysate was similarly applied to the BSA-coupled beads to assess non-specific protein interactions. Gel slices of the protein bands that bound the Ubl beads were compared to the BSA control for protein identification by LC-MS/MS analysis.

### Purification of RecJ3/4-aRNase J and RecJ3/4

For purification of the RecJ3/4-aRNase J complex, the following strains were used: *H. volcanii* HJ02-pJAM4251 expressing His-RecJ3 and *H. volcanii* HJ02- and HJ07-pJAM4252 strains expressing His-RecJ3 and aRNase J-StrepII. HJ02-pJAM202c served as the empty vector control (HJ07-pJAM202c is not viable, as *rnj* is essential). The strains were inoculated into 4 mL ATCC974 medium supplemented with novobiocin (0.3 mg/mL) until OD_600_ 0.8–1.0. Cells were transferred to fresh medium (2–8 × 500–750 mL per 2.8 L Fernbach flask) supplemented with novobiocin and DMSO (100 mM). Cells were cultured at 42°C (200 rpm) to stationary phase with OD_600_ of 1.5–2.0. The cells were harvested by centrifugation at 3,000 × *g* for 20 min at 4°C and stored as pellets at −80°C until used. Cells (3 g wet weight) were resuspended in 15 mL buffer (50 mM HEPES, pH 7.5, 2 M NaCl, 10% glycerol, 1 mM DTT, 4 µg/mL DNase, 5 mM MnCl_2_, EDTA-free protease inhibitor mini tablets, and 40 mM imidazole) and lysed thrice by French press at 2,000 psi (onto ice). Cell lysate was clarified by centrifugation at 13,177 × *g* for 40 min at 4°C and filtration (0.2 µm, cellulose acetate membrane). Cell lysate was applied to HisTrap HP column pre-equilibrated with binding buffer [50 mM HEPES, pH 7.5, 2 M NaCl, 10% (vol/vol) glycerol, 1 mM DTT, 5 mM MnCl_2_, 40 mM imidazole] and washed with the same buffer. His-RecJ3 and its associates were eluted using elution buffer (binding buffer plus 460 mM imidazole) into 1 mL fractions. For strains that also expressed RNaseJ-StrepII, the purified protein was dialyzed against dialysis buffer (50 mM HEPES pH 7.5, 2 M NaCl, 1 mM DTT, 5 mM MnCl_2_, and 5 mM MgCl_2_) and then applied to Strep-Tactin resin pre-equilibrated with binding buffer (50 mM HEPES, pH 7.5, 2 M NaCl, 1 mM DTT, and 5 mM MnCl_2_) by batch purification. The buffers were freshly prepared to avoid MnCl_2_ precipitation. After washing four times with the binding buffer, the RecJ3/4-aRNase J complex was eluted with 1 mL elution buffer (binding buffer plus 5 mM desthiobiotin). His-trap fractions that did not bind the Strep-Tactin resin (flowthrough) were collected as RecJ3/4 subcomplexes. Protein fractions were dialyzed against dialysis buffer supplemented with 10% glycerol and concentrated using Vivaspin 500 centrifugal concentrators (Sartorius, Goettingen, Germany) as needed. RecJ3/4 subcomplexes were further purified by size exclusion chromatography in 50 mM HEPES, pH 7.5, 2 M NaCl, 1 mM DTT, 5 mM MnCl_2_, 5 mM MgCl_2_, and 10% glycerol. Purified proteins were analyzed by reducing 10% SDS-PAGE and nuclease activity assays. Buffers substituted with Tris-Cl and TCEP [tris (2-carboxyethyl) phosphine hydrochloride] for the HEPES and DTT, respectively, were found to yield similar results.

### Purification of aRNase J homodimer

For purification of the aRNase J homodimer, *H. volcanii* HS01-pJAM1406 that expresses aRNase J-StrepII was used. Cultures were initiated by adding 10 mL ATCC974 medium supplemented with novobiocin (0.3 mg/mL) to two plates of isolated colonies and transferring the resuspended cells to a 500-mL Erlenmeyer flask. Culture volume was adjusted to 100 mL final volume with the same medium, and cells were grown to OD_600_ 0.8–1.0. This culture was used as a source of the 10-mL per flask inoculum into fresh ATCC medium (8 × 500 mL per 2.8-L Fernbach flask) supplemented with novobiocin and DMSO (100 mM). Cells were cultured at 42°C (200 rpm) to a stationary phase with OD_600_ of 1.5–2.0. The cells were harvested by centrifugation at 3,000 × *g* for 20 min at room temperature and stored as pellets at −80°C until used. Cells (3 g wet weight) were resuspended in 15 mL buffer (50 mM HEPES, pH 7.5, 2 M NaCl, 1 mM TCEP, 4 µg/mL DNase, 5 mM MnCl_2_, EDTA-free protease inhibitor at 1 tablet/50 mL) and lysed thrice by French press at 2,000 psi (onto ice). Buffers were freshly prepared to avoid MnCl_2_ precipitation. Cell lysate was clarified by centrifugation at 13,177 × *g* for 40 min at 4°C and filtration (0.2 µm, cellulose acetate membrane). Cell lysate was applied to Strep-Tactin resin pre-equilibrated with binding buffer (50 mM HEPES, pH 7.5, 2 M NaCl, 1 mM TCEP, and 5 mM MnCl_2_) and washed with the same buffer. aRNase J-StrepII was eluted using elution buffer (binding buffer plus 5 mM desthiobiotin). Protein fractions were dialyzed against dialysis buffer (50 mM HEPES, pH 7.5, 2 M NaCl, 1 mM DTT, and 5 mM MnCl_2_), concentrated with Vivaspin 500 centrifugal concentrators, and further purified by size exclusion chromatography in the dialysis buffer. Purified protein was analyzed by reducing 10% SDS-PAGE and nuclease activity assay.

### RecJ4 role in RecJ3/4-aRNase J complex assembly

To evaluate the function of RecJ4 in the assembly of the RecJ3/4-aRNase J complex, the tandem expression plasmid pJAM4252 was transformed into HJ04 to simultaneously produce His-RecJ3 and aRNase J in the absence of RecJ4. Purifications were performed successively with HisTrap and Strep-Tactin resin as described above. The host strain HJ04 was constructed by deleting *recJ4* and replacing the His-rich *H. volcanii pitA* with *N. pharonis pitA*.

### Size exclusion chromatography and calibration

Samples were freshly dialyzed in SEC buffer (50 mM HEPES, pH 7.5, 2 M NaCl, 1 mM DTT, 5 mM MnCl_2_, 5 mM MgCl_2_, and 10% glycerol for RecJ3/4, with MgCl_2_ excluded for the aRNase J homodimer). Samples (0.5 mL) were applied at 0.3 mL/min to the Superdex 200 Increase 10/300Gl (SEC) column equilibrated in the same buffer. Fractions (0.5 mL) were collected and analyzed by 10% reducing SDS-PAGE. The SEC column was calibrated in low-salt buffer (50 mM HEPES, pH 7.5, 150 mM NaCl, 1 mM DTT, 5 mM MnCl_2_, 5 mM MgCl_2_, and 10% glycerol) using the following standards: blue dextran (void volume), ferritin (880 and 440 kDa), aldolase (158 kDa), alcohol dehydrogenase (150 kDa), bovine albumin (66 kDa), and thyroglobulin (669 and 1338 kDa) (Sigma).

### Pull-down assays

#### aRNase J pull-down assays

*H. volcanii* H26 “wild-type” (H26, parent) and HS01 (*ΔrecJ3* mutant) strains carrying plasmid pJAM1406 (*rnj-strepII*) and pJAM202c (empty vector control) were used for pull-down assays. Plasmid pJAM1406 was designed to express aRNase J with a C-terminal StrepII tag (-StrepII). Freshly isolated colonies were inoculated into ATCC974 medium supplemented with Nv and 100 mM DMSO (5 mL cultures) for 24 h and then transferred to fresh medium (0.5–1 L per 2.8-L Fernbach flask). Cells were cultured at 42°C (200 rpm). At stationary phase (OD_600_ of 2.5), cells (1 L culture) were harvested by centrifugation (3,000 × *g* for 15 min at 4°C) and washed in ice-chilled buffer (50 mM Tris-HCl, pH 7.5, and 2 M NaCl). Cells were stored as pellets at −80°C until used. Cells (3 g wet weight) were resuspended in 12 mL lysis buffer (50 mM Tris-HCl, pH 7.5, 2 M NaCl, 1 mM DTT, 4 µg/mL DNase, 0.5 mM MnCl_2_, 4.5 mM MgCl_2_, and EDTA-free protease inhibitor mini tablets, Pierce, Thermo Scientific, Waltham, MA, USA) and lysed thrice by French press (2,000 psi). Cell lysate was clarified by centrifugation (9,200 × *g* twice for 15 min at 4°C) and filtration (0.2 µm, cellulose acetate membrane). For the H26 strains, the cell lysate was dialyzed twice for 2 h each time against 4 L buffer (50 mM Tris-HCl, pH 7.5, 2 M NaCl, 1 mM DTT, 0.5 mM MnCl_2_, and 4.5 mM MgCl_2_) at 4°C. The dialyzed cell lysate of the H26 strains was transferred to ice and supplemented with 0.1 mM γATP (adenosine 5′-[γ-thio]triphosphate tetralithium salt, Sigma). For H26 and HS01 strains, the protein samples were applied to Strep-Tactin Superflow Plus resin (2 mL slurry) pre-equilibrated in buffer (50 mM Tris-HCl, pH 7.5, 2 M NaCl, 1 mM DTT, 0.5 mM MnCl_2_, and 4.5 mM MgCl_2_) and washed with the same buffer. For H26 strains, proteins were eluted using an equal volume of 2× SDS-reducing buffer and boiling the Strep-Tactin beads (200 µL resin per liter of culture) for 6 min. Samples were centrifuged at 14,549 × *g* (10 min at 21°C). Proteins, in the supernatant, were separated by reducing 10% SDS-PAGE and analyzed by LC-MS/MS. For the HS01 strains, proteins were eluted from the Strep-Tactin beads using desthiobiotin. Eluted samples were dialyzed and concentrated using Vivaspin 500 centrifugal concentrators (Sartorius, Goettingen, Germany).

#### RecJ4 pull-down assay

The RecJ4 pull-down assay was performed similar to that of aRNase J isolated from H26 with the following exceptions. *H. volcanii* H26-pJAM1405 was used for purification of RecJ4-StrepII. Cell lysate was dialyzed twice for 2 h each time against 4 L buffer (50 mM Tris-HCl, pH 7.6, 2 M NaCl, 1 mM DTT, and 10 mM MgCl_2_) supplemented with 0.1 mM ATP (first dialysis) and 0.1 mM γATP (second dialysis). Protein sample was applied to a Strep-Tactin Superflow Plus resin (2 mL slurry) pre-equilibrated in buffer (50 mM Tris-HCl, pH 7.6, 2 M NaCl, 1 mM DTT, and 10 mM MgCl_2_) and washed with the same buffer prior to elution.

#### Cdc48a pull-down assay

The Cdc48a pull-down assays were as above with the following exceptions. *H. volcanii* H1209-pJAM1409 was used for the purification of His6-Cdc48a. Cells were washed in ice-chilled buffer (10 mM sodium phosphate buffer, pH 7.6, and 2 M NaCl) prior to storing as pellets at −80°C. The lysis buffer was composed of 10 mM sodium phosphate buffer, pH 7.6, 2 M NaCl, 40 mM imidazole, 4 µg/mL DNase, protease inhibitor tablet, 1 mM DTT, 1 mM ATP, and 5 mM MgCl_2_. Protein samples were applied to a His Trap HP column (5 mL, GE Healthcare) pre-equilibrated in the lysis buffer (minus the protease inhibitor and DNase) and washed with this same buffer. The His6-Cdc48a protein was eluted by supplementing the equilibration buffer with 500 mM imidazole. The column eluates were boiled (6 min) in an equal volume of 2× SDS-reducing buffer and centrifuged (14,549 × *g* for 10 min at 21°C).

### Purification of *H. volcanii* RecJ3, RecJ4, and aRNase J proteins from recombinant *E. coli*

*H. volcanii recJ3, recJ4,* and *rnj* genes were subcloned into the plasmid vector pET28b. The *recJ3* and *recJ4* were fused to N-terminal His-tags, while the *rnj* had a C-terminal StrepII tag. Expression plasmids (verified by DNA sequencing) were transformed into *E. coli* Rosetta (DE3) for the synthesis of recombinant proteins. Isopropyl-β-d-1-thiogalactopyranoside (0.5 mM final concentration) was added to flask cultures (OD_600_ of 0.6) to induce recombinant protein expression at 18°C for 16 h. Cells were lysed, the extract was clarified, and proteins were purified similar to the *H. volcanii* purified proteins with the following modifications. PEG 8000 was added to the clarified lysate at a final concentration of 5%, and the protein mixture was gently stirred for 15 min at 21°C. The pellet was discarded after centrifugation at 10,800 × *g* for 15 min at 4°C. The concentration of PEG 8000 in the supernatant was increased to 15%, and the protein fraction was precipitated. After centrifugation at 10,800 × *g* for 15 min, the protein pellet was resuspended in the purification buffer to a final protein concentration of 10 mg/mL. For His-Trap chromatography, the column-bound proteins were further washed with buffer supplemented 60, 80, and 100 mM imidazole before using elution buffer (binding buffer plus 460 mM imidazole) into 1 mL fractions. Purity of proteins was determined by 10% reducing SDS-PAGE.

### Nuclease activity assays

Purified proteins were examined for nuclease activity using 6-carboxyfluorescein (6-FAM) 5′- and 3′-end-labeled oligonucleotide substrates under reaction conditions as detailed in figure legends. The 6-FAM-labeled oligonucleotide substrates (Table S1) were synthesized and desalted by Integrated DNA Technologies (Coralville, IA, USA) with the 3′R substrate requiring RP-HPLC purification prior to assay. Protein concentration for assay was determined by bicinchoninic-acid (BCA) assay, using bovine serum albumin as the standard, according to supplier (Pierce, Thermo Fisher Scientific, Waltham, MA, USA). To quench the nuclease activity assays, samples were rapidly transferred to ice and mixed with an equal volume of 2× loading buffer [90% (vol/vol) formamide, 100 mM EDTA, 0.2% (wt/vol) SDS, 10% (vol/vol) glycerol, and 0.1% (wt/vol) bromophenol blue, and 0.1% (wt/vol) xylene cyanol]. The mixture was heated for 5 min at 92°C, ice chilled for 5 min, and separated by denaturing electrophoresis in 0.5× TBE (Tris-borate-EDTA) buffer (Invitrogen, Thermo Fisher Scientific, Waltham, MA, USA). Gels consisted of 20% or 30% (29:1) acrylamide/bisacrylamide, 8 M urea, and 0.5× TBE buffer and were freshly prepared in an 8 × 10 cm mini vertical format using the Mini-PROTEAN Electrophoresis System (Bio-Rad, Hercules, CA, USA). Gels were subjected to pre-electrophoresis at 25 W for 30 min prior to sample application. Sample separation was at 15 W for 30 min. For further optimization, gels were pre-equilibrated at 30 W for 30 min (for two gels), and samples were separated by electrophoresis at 20 W for 26 min. 6-FAM fluorescence was detected in gel using the iBright FL1000 Imaging System (Thermo Fisher Scientific) at 488 nm excitation with smart exposure program. For enzyme kinetics (performed at least in triplicate), the resulting images were analyzed using ImageJ 1.53s software according to provider (112). *V*_max_ and *k*_cat_ calculations were performed by plotting data in Microsoft Excel. All results were found experimentally reproducible.

### LC-MS/MS analysis

For analysis from gel slices, protein samples were separated by 10% SDS-PAGE, visualized by staining with Bio-Safe Coomassie (Bio-Rad, Hercules, CA, USA), and destained in double deionized water. SDS-PAGE gels were under reducing conditions with the exception of the Ubl pull-down samples, which were analyzed under non-reducing conditions to examine non-covalent interactions. Unique protein bands were excised and analyzed by LC-MS/MS. Equivalent regions of the gel were similarly analyzed for the empty vector control. Proteins in the gel slices were treated with 45 mM DTT and 100 mM 2-chloroacetamide (CAA). To minimize CAA carryover prior to trypsin digest, liquid was removed from the treated gel pieces, and samples were washed with 25 mM ammonium bicarbonate buffer (pH > 7.9), dehydrated by treatment with acetonitrile, and treated by centrifugal evaporation (SpeedVac, Labconco, Kansas City, MO, USA) to dryness. Samples were treated with trypsin (1 µg trypsin per 50 µg protein) at 37°C for 15 h. Tryptic peptides were injected onto a capillary trap (Thermo Scientific PepMap) and desalted for 5 min with 0.1% (vol/vol) formic acid at a flow rate of 3 µL/min prior to loading onto a Thermo Scientific C18 Pep Map nanoflow high-performance liquid chromatography (HPLC) column. The elution gradient of the HPLC column started at 3% solvent A [0.1% (vol/vol) formic acid, 3% (vol/vol) acetonitrile, and 96.9% (vol/vol) H_2_O], 97% solvent B [0.1% (vol/vol) formic acid, 96.9% (vol/vol) acetonitrile, and 3% (vol/vol) H_2_O] and finished at 60% solvent A, 40% solvent B using a flow rate of 300 nL/min for 30 min. LC-MS/MS analysis of the eluting fractions was carried out on an LTQ Orbitrap XL mass spectrometer (Thermo Scientific, San Jose, CA, USA). The instrument under Xcalibur 2.07 with LTQ Orbitrap Tune Plus 2.55 software was operated in the data-dependent mode to automatically switch between MS and MS/MS acquisition. Full MS scans were acquired with a resolution of 60,000 in the Orbitrap from *m*/*z* 300–2,000. Ten most intense ions were fragmented by collision-induced dissociation (CID) at a target value of 5,000 or maximum ion time of 150 ms. Dynamic exclusion was set to 60 s. Typical mass spectrometric conditions include a spray voltage of 2.2 kV, no sheath and auxiliary gas flow, a heated capillary temperature of 200°C, a capillary voltage of 44 V, a tube lens voltage of 165 V, an ion isolation width of 1.0 *m*/*z*, and a normalized CID collision energy of 35% for MS2 in linear ion trap. The ion selection threshold was 500 counts for MS2. An activation *q* = 0.25 and activation time of 30 ms were set. Raw data were analyzed using Mascot (Matrix Science, London, UK; version 2.2.2) against *H. volcanii* (Uniref UP000008243) and target decoy databases with the latter, including a set of reversed sequences generated by Mascot. Mascot was searched with a fragment ion mass tolerance of 0.8 Da and a parent ion tolerance of 15 ppm. Carbamidomethylation of Cys was indicated as a fixed modification, while deamidation of Asn and Gln, oxidation of Met, and isopeptide linkage to Gly-Gly- were specified as variable modifications. Scaffold (Proteome Software Inc., Portland, OR, USA) was used to validate MS/MS-based peptide and protein identifications, where protein probabilities were assigned by the Protein Prophet algorithm, and peptide probabilities were assigned by the Peptide Prophet algorithm (113, 114). Protein identities and diglycine footprints were based on a threshold of 99.9% probability and <0.1% FDR and are summarized in Data set S1. Normalized total spectral counts were calculated using the quantitative package associated with Scaffold (115).

### RecJ3/4-aRNAse J subunit stoichiometry

The subunit stoichiometry of the RecJ3/4-aRNase J complex purified by TAP from HJ02-pJAM4252 was analyzed by absolute quantification (AQUA)-based mass spectrometry. Peptides specific to tryptic fragments of aRNase J (382-IYDEIHVSGHLR-393), RecJ3 (137-QTGGPTVFR-145), and RecJ4 (286-AYPEVEVGDYVR-297) were synthesized with stable isotopes, where R represents arginine (^13^C_6_,^15^N_4_) for each peptide. The labeled peptides were used as internal standards, and the absolute levels of the proteins were measured using selected reaction monitoring (SRM) in tandem mass spectrometry (MS/MS) analysis (Data set S1D) (116). Density scan was also used to estimate subunit stoichiometry. Proteins were separated by reducing 10% SDS-PAGE stained in gel using SYPRO-Ruby or Coomassie Blue R-250 and visualized using an iBright FL1000 Imaging System. Protein band density was used to estimate protein quantity using ImageJ 1.53s software (112). The subunit stoichiometries determined by AQUA MS and ImageJ analysis were found comparable.

### AQUA-based MS analysis

Protein concentration was determined by Bradford assay with bovine serum albumin as the standard. Protein (30 µg) was reduced with 40 mM DTT, alkylated with 100 mM of CAA, and digested with trypsin/Lys-C protease mix [Promega Corporation, Madison, WI, USA; at an enzyme to protein ratio (wt/wt) of 1:100]. The tryptic digests were desalted using micro ZipTip C-18 mini-reverse phase according to the manufacture manual (MilliporeSigma, Burlington, MA, USA). In brief, after wetting (with 50% acetonitrile) and equilibrating a ZipTip pipet tip (with 0.1% formic acid), the peptides were bound to C-18 material. A subsequent washing step with 0.1% formic acid was followed, and final elution was carried out with 80% acetonitrile and 0.1% formic acid. The samples were lyophilized to dryness at 160 mBar using a Labconco SpeedVac (Centrivap, Labconco Inc., USA). The resulting peptide pellets were stored at −20°C until targeted MS analysis. A targeted method was developed for SRM to determine the absolute amount of protein in the sample on a TSQ Altis triple quadrupole mass spectrometer interfaced with a nano Easy1200 ultra-performance liquid chromatography (Thermo Scientific, San Jose, CA, USA). Three unique peptides with heavy isotope were synthesized (Thermo Scientific, Pierce Biotechnology, Rockford, IL, USA), and each peptide optimized three transitions. The flow rate was set at 350 nL/min with solvent A (0.1% formic acid in water) and solvent B (0.1% formic acid and 99.9% acetonitrile) as the mobile phases. Separation was conducted using the following gradient: 2%–50% of B over 0–23 min and 50%–98% of B over 23–25 min, holding 98% of B over 25–27 min, and then from 90% to 0% of B from 27 to 28 min. The equilibration at 2% B was from 28 to 30 min. SRM conditions for peptides including *m*/*z* of SRM pairs (precursor and product pairs), collision energy, and RF lens voltage were optimized by direct infusion of the compounds into the MS instrument at a concentration of 500 pg/mL, and the resulting optimized conditions are shown in Data set S1D. The Q1 and Q3 resolutions were 0.7 mass units full width at half maximum. The dwell time of each SRM transition was 15.3 ms. The positive voltage on spray, ion transfer tube temperature, and CID gas (mTorr) were set at 2.4 kV, 325°C, and 1.5, respectively. Calibration standards were prepared by spiking the appropriate quantity of three heavy peptides to final concentrations of 40, 80, 120, 160, 200, 240, 280, 320, 400, 1,000, 5,000, and 8,000 pg/mL. Heavy peptides were spiked to a final concentration of 400 pg/mL. Data processing was carried out using the Quan Brower module of the Xcalibur software ver. 4.1 (Thermo Scientific, San Jose, CA, USA) based on the calibration standards. Data processing was carried out using the Quan Brower module of the Xcalibur program (Thermo Scientific, San Jose, CA, USA), and extracted-ion chromatograms of the peak area from standards were extracted and generated using the calibration standards. The linear regression analysis was used for both constructions of the calibration curve and sample quantification.

### SDS-PAGE and immunoblotting analysis

Protein samples were mixed with an equal volume of 2× SDS-PAGE loading buffer [100 mM Tris-HCl buffer at pH 6.8 with 4% (wt/vol) SDS, 20% (vol/vol) glycerol, 0.6 mg/mL bromophenol blue, and 5% (vol/vol) β-mercaptoethanol]. β-Mercaptoethanol was excluded for non-reducing SDS-PAGE. Samples were boiled for 5–10 min and centrifuged at 12,000 × *g* prior to separation by SDS-PAGE. Separated proteins were electroblotted from the gels onto Immobilon-FL PVDF membranes (0. 45 µm) (MilliporeSigma, Burlington, MA, USA) as per standard protocol (BioRad, Hercules, CA, USA). HRP-conjugated anti-His mouse monoclonal antibody (Proteintech Group Inc, Rosemont, IL, USA) and HRP-conjugated anti-StrepII monoclonal antibody (IBA Lifesciences, Germany) were used for immunoblotting analysis of His-tag and StrepII-tag, respectively. To detect Cdc48a, polyclonal anti-VCP primary antibodies (AbCam product no. ab138298) and alkaline phosphatase (AP)-linked goat anti-rabbit IgG(H + L) secondary antibodies (SouthernBiotech, Birmingham, AL, USA) were used. AP activity was detected using CDP-Star (Applied Biosystems, Framingham, MA, USA). HRP activity was detected using ECL Plus immunoblotting substrate (Pierce, ThermoFischer Scientific, Waltham, MA, USA). Chemiluminescent signals were documented using X-ray film (Amersham Hyperfilm; Cytiva, Marlborough, MA, USA) or an iBright FL1000 (Thermo Fisher Scientific, Waltham, MA, USA).

### Analysis of genomic co-occurrence

InterPro categories were combined to define the RecJ3/4 homologs as requiring classification to the DHH phosphoesterase superfamily (IPR038763), the nucleic acid-binding OB-fold superfamily (IPR012340), and RNA-binding S1 domain (IPR022967). aRNase J homologs were of the aRNase J family (IPR004613) and were restricted to archaea. Similar restrictions were used to define RNA degradosomes, RNA exosomes, and other related homologs as summarized in Table S2 and Data Set S2. Protein lists were downloaded from UniProtKB/TrEMBL using the advanced search engine (https://www.uniprot.org/) (117). To generate genome neighborhood networks (GNNs), protein lists were first entered as accession IDs into the Enzyme Similarity Tool (EFI-EST) web portal (https://efi.igb.illinois.edu/efi-est/) (118). The resulting data set was assessed to determine the alignment score that would generate an SSN at 40%–50% amino acid sequence identity. This score was used to finalize and enter the SSN into the Genome Network Tool (EFI-GNT) for query by Pfam numbers to assess the co-occurrence of homologs within target protein (RecJ3, RecJ4, and aRNase J)-defined genome neighborhoods. For Cdc48a and uncharacterized proteins of interest, the *H. volcanii* protein sequences (HVO_2380, HVO_2382, and HVO_1964) were entered into the sequence blast option of the EFI-EST web portal for generating the SSN used for GNN analysis. The criteria for inclusion in the GNN were that the homologs were detected within a window of 10 genes to occur in at least 70 archaeal genomes (with an exception of the RecJ3-Cdc48 synteny noted in 26 methanogen genomes).

## Data Availability

Proteomic data sets are available through the PRoteomics IDEntifications (PRIDE) database (119) under accession numbers PXD019896, PXD019897, PXD019898, PXD019906, PXD019931, PXD019932 , and PXD019933.

## SUPPLEMENTAL MATERIAL

Data Set S1-A-G mbio.00852-23-s0001.xlsx Data associated with the identification and characterization of the Ubl interactome including RecJ3, RecJ4, aRNase J, and Cdc48a.

Data Set S1-H-K mbio.00852-23-s0002.xlsx Data associated with the identification and characterization of the Ubl interactome including RecJ3, RecJ4, aRNase J, and Cdc48a.

Data Set S2 mbio.00852-23-s0003.xlsx Data associated with genome neighborhood networks and co-occurrence patterns.

Figure S1 mbio.00852-23-s0004.pdf Deletion of *rnj* (aRNase J) gene in *H. volcanii* H26 carrying plasmid pJAM4253 (*rnj*^+^).

Figure S2 mbio.00852-23-s0005.pdf Reducing SDS-PAGE gels of RecJ3, RecJ4, aRNase J, and Cdc48a proteins purified from recombinant *E. coli* and *H. volcanii* strains (that are in addition to Fig. 4).

Figure S3 mbio.00852-23-s0006.pdf Immunoblotting analysis reveals multiple bands of Cdc48a consistent with its Ubl-modification.

Figure S4 mbio.00852-23-s0007.pdf Examples of genome neighborhoods of archaeal RecJ3, RecJ4, aRNase J, and Cdc48a gene homologs.

Figure S5 mbio.00852-23-s0008.pdf Cdc48a, RecJ3, RecJ4, and aRNase J homologs analyzed by 3D-structural modeling, protein domain analysis, and multiple amino acid sequence alignment.

Table S1 mbio.00852-23-s0009.docx List of plasmids, strains and primers used in this study.

Table S2 mbio.00852-23-s0010.docx Genome synteny of RecJ4, RecJ3, aRNase J, and Cdc48a homologs in archaea.

ASM does not own the copyrights to Supplemental Material that may be linked to, or accessed through, an article. The authors have granted ASM a non-exclusive, world-wide license to publish the Supplemental Material files. Please contact the corresponding author directly for reuse.
