# Supplementary material for: RecJ3/4-aRNase J form a Ubl-associated nuclease complex functioning in survival against DNA damage in Haloferax volcanii
Source: mBio. 2023 Jul 17;14(4):e00852-23. doi: 10.1128/mbio.00852-23 (PMC10470531; doi:10.1128/mbio.00852-23)
Supplement: Figure S1 — Deletion of rnj (aRNase J) gene in H. volcanii H26 carrying plasmid pJAM4253 (rnj+). [file mbio.00852-23-s0004.pdf]

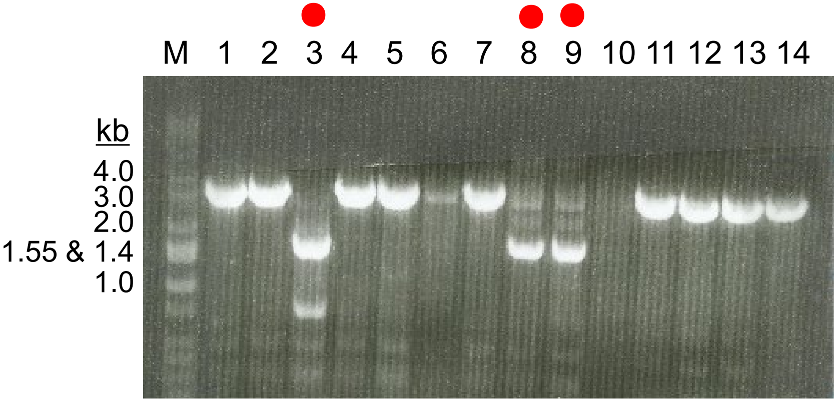

Figure S1. Deletion of *rnj* (RNase J) gene in *H. volcanii* H26 carrying plasmid pJAM4253 (*rnj*+). PCR screening of the *Δrnj* deletion in *H. volcanii* H26 carrying plasmid pJAM4253 (*rnj*+). Lane 1-13, single colony strains screened. Lane 14, H26-pJAM4253 parent that was not subjected to homologous recombination. HJ06 strains with the *Δrnj* deletion are indicated by ●. Plasmid pJAM4253 could not be cured from the HJ06 *Δrnj* mutant strains. When using the same *Δrnj* deletion plasmid (pJAM4261), the *Δrnj* mutation could not be generated in the H26 strain alone or in H26 strains carrying the empty vector (pJAM202c).
