# Supplementary material for: RecJ3/4-aRNase J form a Ubl-associated nuclease complex functioning in survival against DNA damage in Haloferax volcanii
Source: mBio. 2023 Jul 17;14(4):e00852-23. doi: 10.1128/mbio.00852-23 (PMC10470531; doi:10.1128/mbio.00852-23)
Supplement: Figure S2 — Reducing SDS-PAGE gels of RecJ3, RecJ4, aRNase J, and Cdc48a proteins purified from recombinant E. coli and H. volcanii strains (that are in addition to Fig. 4). [file mbio.00852-23-s0005.pdf]

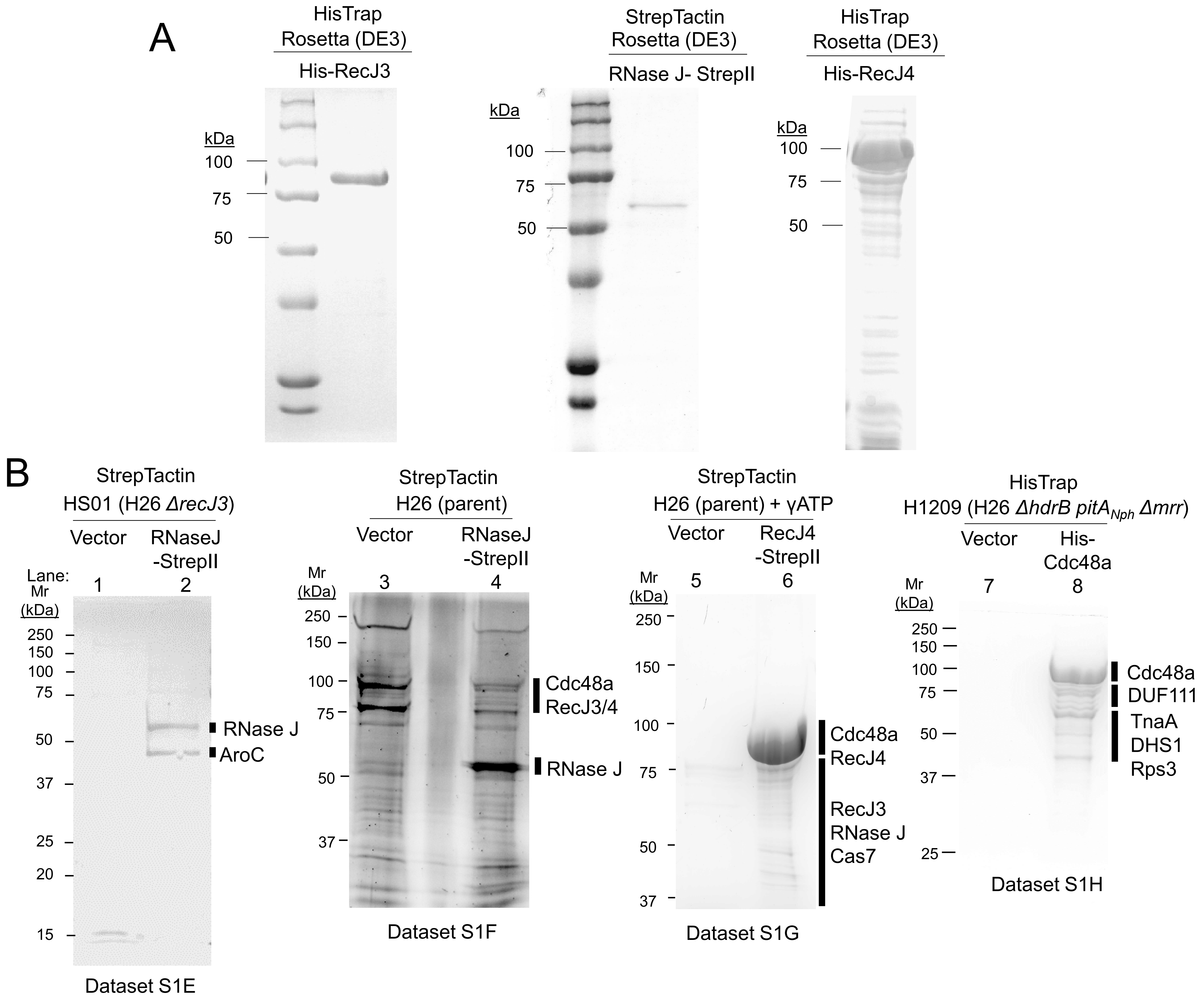

Figure S2. Reducing SDS-PAGE gels of RecJ3, RecJ4, RNase J, and Cdc48a proteins purified from recombinant *E. coli* (A) and *H. volcanii* (B). The affinity tagged proteins were expressed and purified by StrepTactin (RNase J-StrepII and RecJ4-StrepII) or HisTrap (His-RecJ3, His-RecJ4 and His-Cdc48a) chromatography from the host strains as indicated. For purification of His-RecJ4 from *E. coli,* the flowthrough fractions from the His-Trap column were found to contain the soluble protein. All other proteins were found to bind the affinity column. For purification of RecJ4-StrepII from *H. volcanii*, the buffers were supplemented with γATP as indicated and detailed in methods. Vector, indicates the samples similarly purified from the *H. volcanii* host strain carrying the pJAM202c empty vector control. Purified proteins were separated by reducing SDS-PAGE and stained with Coomassie blue, SyproRuby or BioSafe Coomassie blue. Black bars on right of gels in panel B indicate regions where the gel slices were excised and analyzed by LC-MS/MS. Proteins indicated on right were identified by LC-MS/MS analysis based on a high score (SEQUEST HT or Normalized Total Spectra score) and FDR < 0.01%, protein threshold > 99%, 2 peptide minimum. For supporting details see **Datasets S1E-H** as indicated.
