# Supplementary material for: RecJ3/4-aRNase J form a Ubl-associated nuclease complex functioning in survival against DNA damage in Haloferax volcanii
Source: mBio. 2023 Jul 17;14(4):e00852-23. doi: 10.1128/mbio.00852-23 (PMC10470531; doi:10.1128/mbio.00852-23)
Supplement: Figure S3 — Immunoblotting analysis reveals multiple bands of Cdc48a consistent with its Ubl-modification. [file mbio.00852-23-s0006.pdf]

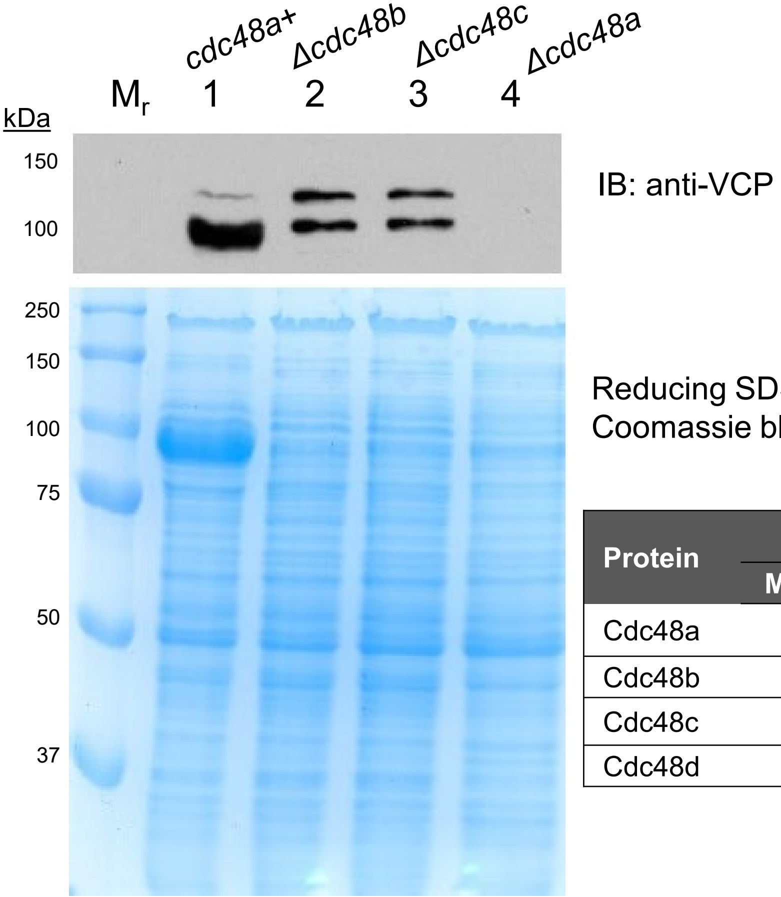

Protein	Theoretical	
	M_r (kDa)	pI
Cdc48a	82.0	4.71
Cdc48b	82.8	4.63
Cdc48c	78.2	4.76
Cdc48d	53.4	4.42

Figure S3. Immunoblotting analysis reveals multiple bands of Cdc48a consistent with its Ubl-modification. *H. volcanii* cells (OD_{600} of 0.08 units per lane) were lysed in reducing SDS-PAGE sample buffer by boiling 5-10 min. After cooling, the samples were separated by 10% reducing SDS-PAGE and analyzed by immunoblotting with anti-VCP antibody (AbCam product no. ab138298). Lane 1, H26-pJAM1400 (Cdc48a-StrepII ectopically expressed). Lane 2, NN2 (*Δcdc48b*). Lane 3, NN3 (*Δcdc48c*). Lane 4, H1999 (*Δcdc48a*).
