# Supplementary material for: RecJ3/4-aRNase J form a Ubl-associated nuclease complex functioning in survival against DNA damage in Haloferax volcanii
Source: mBio. 2023 Jul 17;14(4):e00852-23. doi: 10.1128/mbio.00852-23 (PMC10470531; doi:10.1128/mbio.00852-23)
Supplement: Figure S4 — Examples of genome neighborhoods of archaeal RecJ3, RecJ4, aRNase J, and Cdc48a gene homologs. [file mbio.00852-23-s0007.pdf]

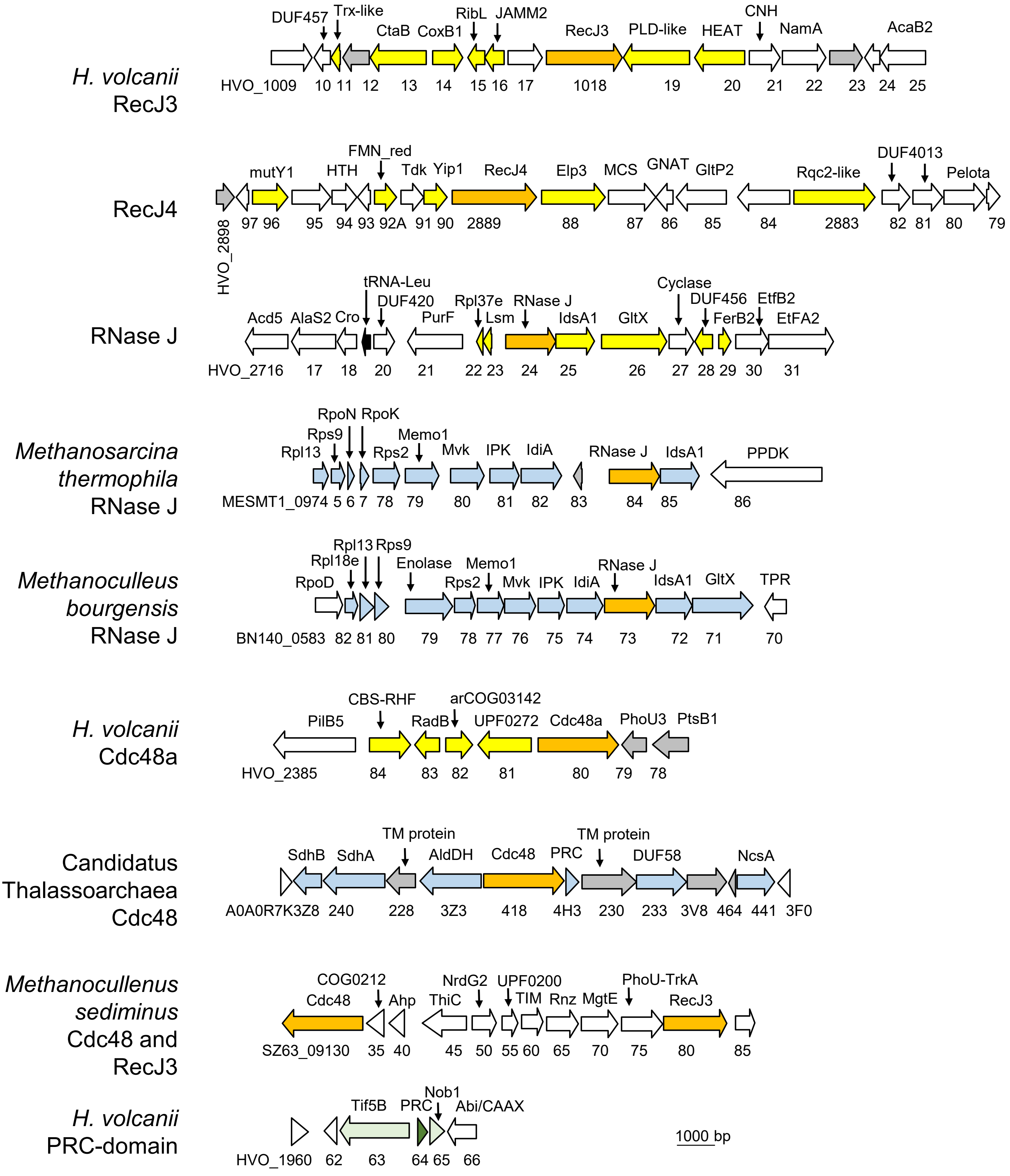

Figure S4. Examples of genome neighborhoods of archaeal RecJ3, RecJ4, RNase J and Cdc48a gene homologs. Orange, Cdc48a, RecJ3/4 and RNase J used as ‘targets’; yellow and blue, homologs in synteny with ‘targets’ in at least 70 other archaeal genomes; grey, no distinct Pfam or InterPro classification, thus, limiting estimation of co-occurrence by GNN analysis; dark and light green, *H. volcanii* PRC, Nob1 and Tif5B gene homologs. See **Table S1** and **Dataset S2B** for details and abbreviations.
