# Supplementary material for: RecJ3/4-aRNase J form a Ubl-associated nuclease complex functioning in survival against DNA damage in Haloferax volcanii
Source: mBio. 2023 Jul 17;14(4):e00852-23. doi: 10.1128/mbio.00852-23 (PMC10470531; doi:10.1128/mbio.00852-23)
Supplement: Figure S5 — Cdc48a, RecJ3, RecJ4, and aRNase J homologs analyzed by 3D-structural modeling, protein domain analysis, and multiple amino acid sequence alignment. [file mbio.00852-23-s0008.pdf]

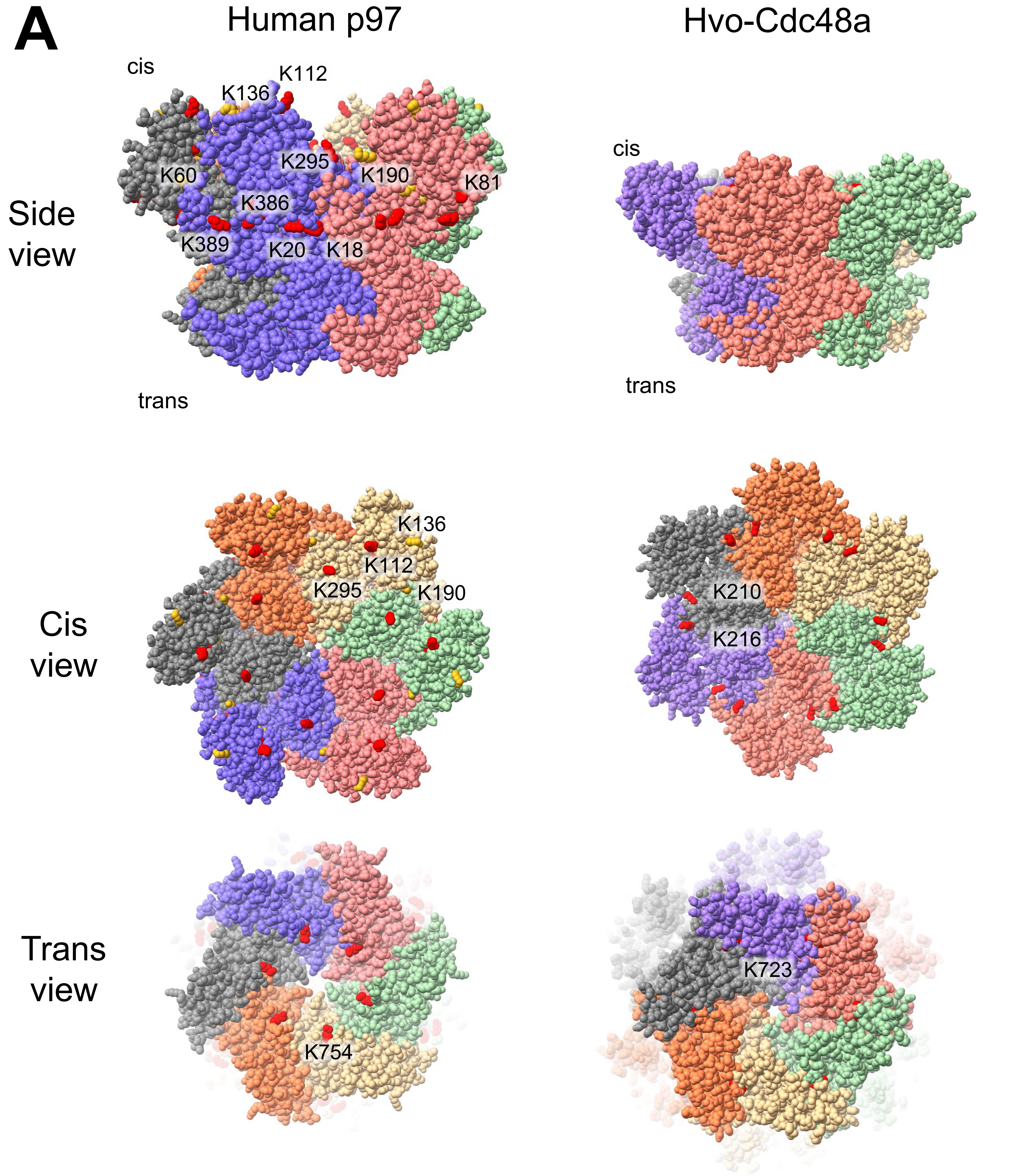

Figure S5. Cdc48a, RecJ3, RecJ4 and aRNase J homologs analyzed by 3D-structural modeling, protein domain analysis and multiple amino acid sequence alignment.

Fig. S5A. Comparison of Ub/Ubl-modification sites of *H. volcanii* (Hvo) Cdc48a to human p97. Cryo-EM structure of ATPγS bound human p97 (PDB: 5FTN, left panels) and Phyre2-generated 3D-homology model of *H. volcanii* Cdc48a (right panels) displayed in hexameric configuration. Ub/Ubl-modification sites are displayed in red and yellow spheres with lysine position numbers indicated. Cis, indicates the interface that engages in the unraveling of protein substrates. Trans, designates the surface that harbors a portal where protein substrates exit the complex in an unfolded state. For supporting details see **Dataset S1H**.

B

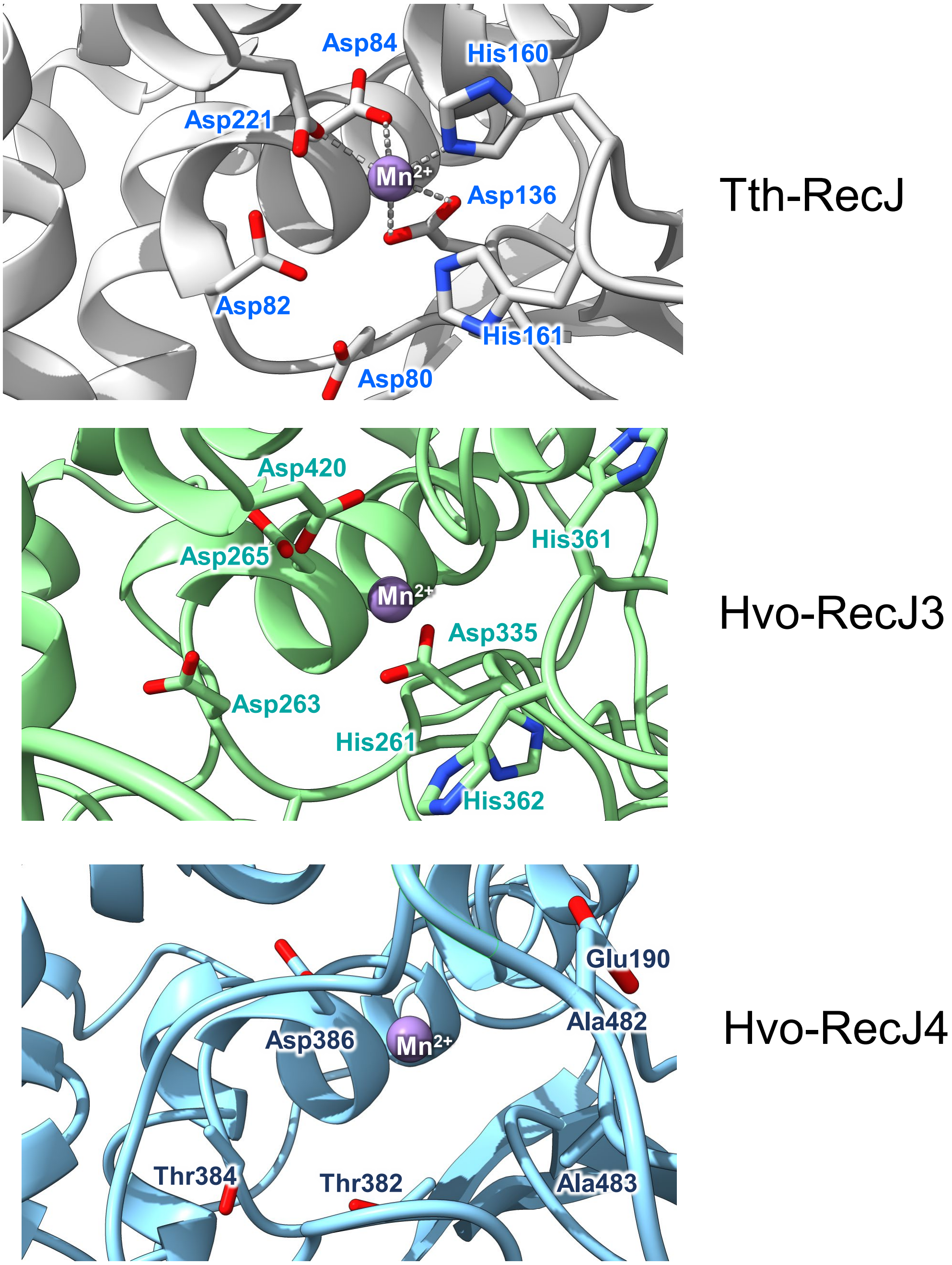

*Tt*RecJ	*Hv*RecJ3	*Hv*RecJ4
His161	His362	Ala483
**His160**	**His361**	Ala482
**Asp221**	**Asp420**	x
**Asp84**	**Asp265**	**Asp386**
**Asp82**	**Asp263**	Thr384
**Asp80**	His261	Thr382
**Asp136**	**Asp335**	x

Figure S5B. Comparison of *H. volcanii* RecJ3 and RecJ4 to *Thermus thermophiles* (Tth) RecJ exonuclease. *H. volcanii* (Hvo) Hvo-RecJ3 (HVO_1018) and Hvo-RecJ4 (HVO_2889) 3D-structural models were generated by Phyre2-based homology modeling and compared to the X-ray crystal structure of Tth-RecJ (PDB: 1IR6, chain A). Table inset: conserved active site residues that may coordinate the catalytic Mn^{2+} ion (red) and residues suggested to bind a second metal ion in the presence of ssDNA or nucleotide (brown). Residues are also highlighted on the ribbon diagrams.

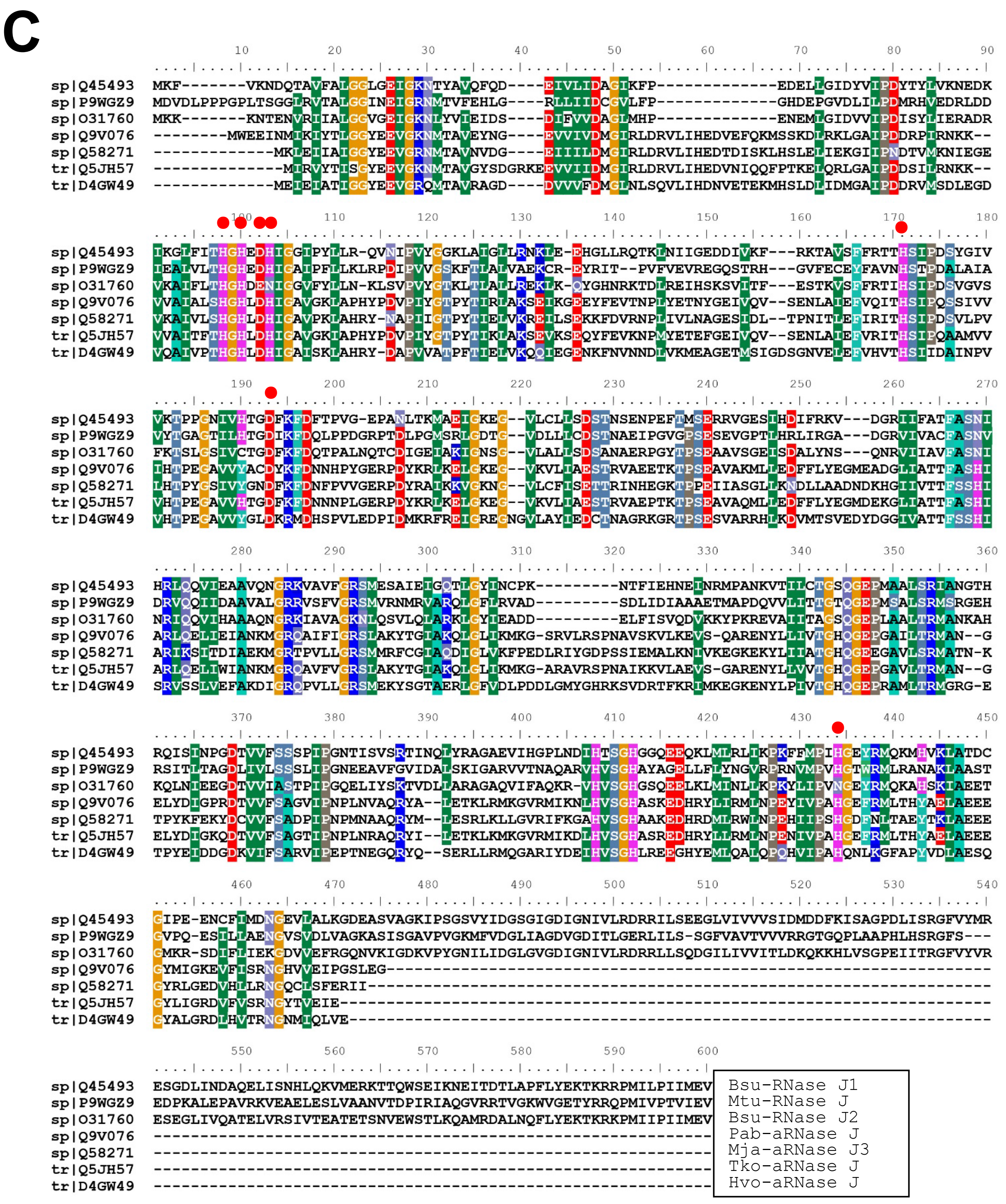

Figure S5C. Multiple amino acid sequence alignment of bacterial and archaeal aRNase J family proteins. UniProtKB numbers (left): *Bacillus subtilis* Bsu-RNase J1/2 (BsRnj1/2, Q45493/O31760), *Mycobacterium tuberculosis* Mtu-RNase J (P9WGZ9), *Pyroccous absysii* Pab-aRNase J (Q9V076), *Methanocaldococcus jannaschii* Mja-aRNase J3 (Q58271), *Thermococcus kodakarensis* Tko-aRNase J (Q5JH57), and *Haloferax volcanii* Hvo-aRNase J (D4GW49). ●, conserved active site residues that coordinate two divalent metal ions (Zn^{2+} or Mg^{2+}) for catalysis. The C-terminal tail common to the bacterial RNase J is not conserved in the archaeal enzymes.

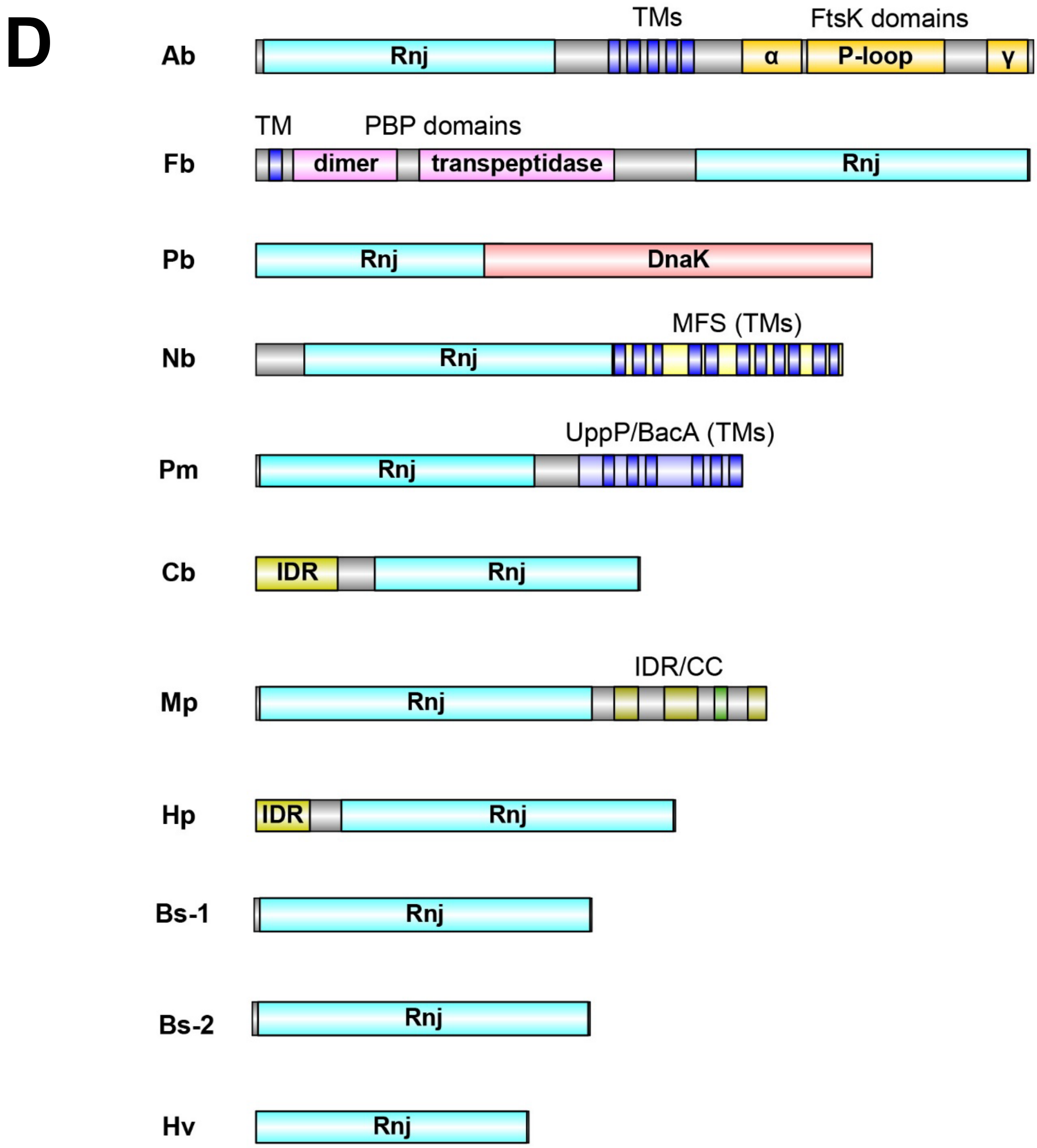

Figure S5D. RNase J family protein domain architecture. Domains/families (InterPro): Rnj, RNase J family (IPR004613); TM, transmembrane domains; FtsK α, P-loop and γ domains (IPR041027, IPR002543 and IPR018541); PBP, penicillin-binding protein domains (IPR005311 and IPR036138), DnaK chaperone family (IPR012725), MFS, major facilitator superfamily (IPR011701); UppP/BacA, undecaprenyl-diphosphatase (IPR003824); IDR, intrinsically disordered region; CC, coiled coil. RNase J homologs (Uniprot): Ab, *Actinobacteria* (A0A537ZKW0); Fb, *Fusobacterium* (G6C271); Pb, *Parcubacteria* (A0A0G1TWX8); Nb, *Nomurabacteria* (A0A3D5Y638); Pm, *Planctomycetales* (A0A1V4QR24); Cb, *Corynebacterium bovis* (A0A426Q1M6); Mp, *Mycoplasma ovipneumoniae* (A0A449AXP7); Hp, *Helicobacter pylori* (B9XZG7); Bs-1/2, *Bacillus subtilis* (Q45493/O31760); Hv, *Haloferax volcanii* (D4GW49).

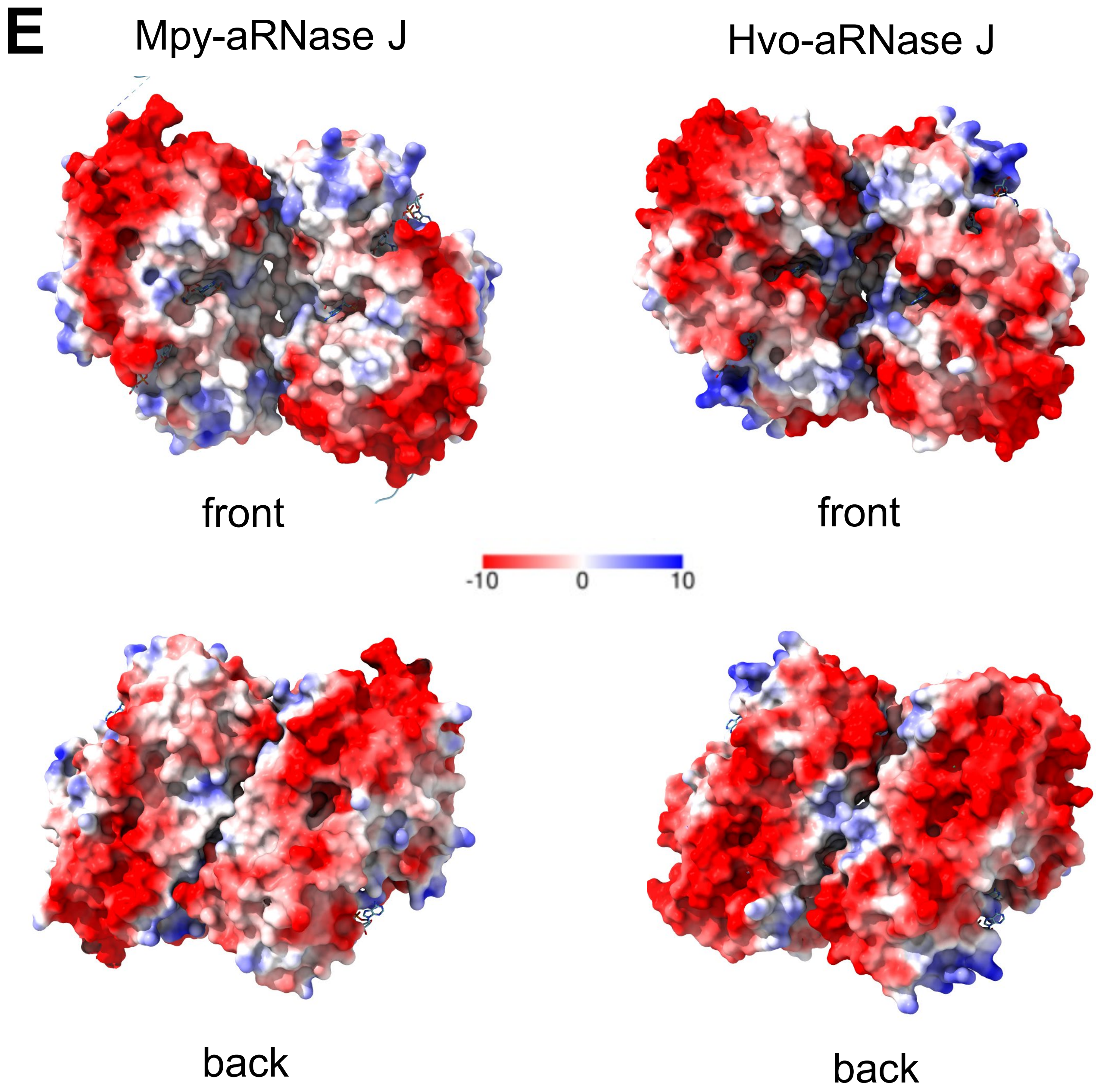

Figure S5E. 3D-structural comparison of *Haloferax volcanii* Hvo-aRNase J and *Methanolobus psychrophilus* Mpy-aRNase J. Surface representation colored according to Coulombic surface charge. The molecules on the bottom ('back') are rotated 180° compared to the top ('front') to provide a more complete visualization of the surface charge. Hvo-aRNase J, 3D-structural model Alpha-fold AF-D4GW49-F1. Mpy-aRNase J, X-ray crystal structure PDB: 6LLB of enzyme with S247A variant in complex with 6 nt RNA, the latter in stick diagram.
