# Supplementary material for: RecJ3/4-aRNase J form a Ubl-associated nuclease complex functioning in survival against DNA damage in Haloferax volcanii
Source: mBio. 2023 Jul 17;14(4):e00852-23. doi: 10.1128/mbio.00852-23 (PMC10470531; doi:10.1128/mbio.00852-23)
Supplement: Table S1 — List of plasmids, strains and primers used in this study. [file mbio.00852-23-s0009.docx]

**Table S1.** List of plasmids, strains and primers used in this study.

| **Strains or plasmid** | **Genotype and/or Description^a^** | | **Ref. or Source** |
| --- | --- | --- | --- |
|  | | |  |
| ***E. coli* strains:** | | |  |
| TOP10 | F^–^ *recA1 endA1 hsdR17*(r_K_^–^ m_K_^+^) *supE44 thi-1 gyrA relA1* | | Invitrogen |
| GM2163 | F^–^ *ara-14 leuB6 fhuA31 lacY1 tsx78 glnV44 galK2 galT22 mcrA dcm-6 hisG4 rfbD1 rpsL136 dam13*::Tn*9 xylA5 mtl-1 thi-1 mcrB1 hsdR2* | | New England Biolabs |
| Rosetta (DE3) | F- *ompT hsdSB*(r_B_- m_B_-) *gal dcm* (DE3) pRARE (Cm^r^) | | Novagen |
|  | | |  |
| ***H. volcanii* strains:** | | |  |
| DS70 | wild-type isolate DS2 cured of plasmid pHV2 | | (1) |
| H26 | DS70 *ΔpyrE2* | | (2) |
| H1209 | H26 *ΔhdrB pitA_Nph_ Δmrr* | | (3) |
| H164 | *ΔpyrE2 ΔtrpA leuB-Ag1 bgaHa-Bb* | | This study |
| H1999 | H164 *Δcdc48a* | | This study |
| H4192  HM1052 | H164 ∆*recJ3 ∆recJ4*  H26 *ΔubaA* | | This study  (4) |
| HM1096 | H26 *Δsamp1 Δsamp2 Δsamp3* | | (4) |
| NH02 | H26 *Δsamp1 Δsamp2 Δsamp3 ΔubaA* | | (5) |
| HS01 | H26 *ΔrecJ3* | | This study |
| HJ02 | H26 *ΔrecJ3 pitA_Nph_* | | This study |
| HJ03 | H26 *ΔrecJ4* | | This study |
| HJ04 | H26 *ΔrecJ4 pitA_Nph_* | | This study |
| HJ05 | H26 T7-*p.tnaA:rnj* by replacing the native promoter of *rnj* | | This study |
| HJ06 | H26:pJAM4253:*Δrnj* | | This study |
| HJ07 | HJ02:pJAM4252:*Δrnj* | | This study |
|  |  | |  |
| **Plasmids:** |  | |  |
| pTA131 | Ap^r^; pBluescript II with *p.fdx*:*pyrE2* | | (2) |
| pJAM202c | Ap^r^; Nv^r^; *Hfx. volcanii*-*E. coli* shuttle plasmid with ColE1 *ori* and pHV2 *ori* | | (6) |
| pJAM503 | Ap^r^;Nv^r^: pJAM202c with *p2.rrn*:*his6- tag* | | (6) |
| pJAM809 | Ap^r^;Nv^r^: pJAM202c with *p2.rrn*:*-strepII tag* | | (7) |
| pTA1106 | Ap^r^; pTA131 with *pitA_Nph_* gene replacement | | (3) |
| pJAM957 | Ap^r^;Nv^r^: pJAM202c with *p2.rrn*:*ubaA-strepII* | | (4) |
| pJAM1131 | Cm^r^, Km^r^, *pT7:flag-his6-samp1* in pET24b | | (8) |
| pJAM4251 | Ap^r^;Nv^r^; pJAM202c with *p2.rrn*:*his6-recJ3* | | This study |
| pJAM4252 | Ap^r^;Nv^r^; pJAM202c with *p2.rrn*:*his6-recJ3*, *p.fdx*:*rnj-strepII* | | This study |
| pJAM4253 | Ap^r^;Nv^r^; pJAM202c with *p2.rrn*:*rnj* | | This study |
| pJAM4254 | Ap^r^;Nv^r^; pJAM202c with *p2.rrn*:*recJ3* | | This study |
| pJAM4255 | Ap^r^; pTA131-based pre-knockout plasmid for HS01 (*ΔrecJ3*) | | This study |
| pJAM4256 | Ap^r^; pTA131-based knockout plasmid for HS01 (*ΔrecJ3*) | | This study |
| pJAM4257 | Ap^r^; pTA131-based pre-knockout plasmid for HJ03 (*ΔrecJ4*) | | This study |
| pJAM4258 | Ap^r^; pTA131-based knockout plasmid for HJ03 (*ΔrecJ4*) | | This study |
| pJAM4259 | Ap^r^; pTA131-based plasmid with T7term-p.*tna*A:*rnj* for HJ05 | | This study |
| pJAM4260 | Ap^r^; pTA131-based pre-knockout plasmid for HJ06 (*Δrnj*) | | This study |
| pJAM4261 | Ap^r^; pTA131-based knockout plasmid for HJ06 (*Δrnj*) | | This study |
| pTA1515  pTA1927  pTA1894  pJAM1405 | Ap^r^; pTA131-based knockout plasmid for H1999 (*Δcdc48a*)  Ap^r^; pTA131-based knockout plasmid for H4192 (∆*recJ3*)  Ap^r^; pTA131-based knockout plasmid for H4192 (∆*recJ4*)  Ap^r^;Nv^r^; pJAM809 with *p2.rrn*:*recJ4-strepII* | | This study  This study  This study  This study |
| pJAM1406 | Ap^r^;Nv^r^; pJAM809 with *p2.rrn*:*rnj-strepII* | | This study |
| pJAM1407 | Ap^r^;Nv^r^; pJAM809 with *p2.rrn*:*recJ3-strepII* | | This study |
| pJAM1409 | Ap^r^;Nv^r^; pJAM503 with *p2.rrn*:*his6*-*cdc48a* | | This study |
| pJAM1410 | Apr;Nvr: pJAM202 with *p2.rrn*:*cdc48a* | | This study |
|  | |  |  |

| **Primer name** | **Primer sequence (5’-3’)** | **Construct** |
| --- | --- | --- |
|  |  |  |
| **Cdc48A:** |  |  |
| 2380_NdeI | 5’-CGACGGCCATATGAACGAAGTCCAACTCGA  AGTGGCGAAAGC-3’ | pJAM1409 |
| HVO_2380 BlpI  Cdc48aBglF  Cdc48aBglR | 5’-AGCTGAGCTTACTGGAAGCCGATGCGG-3’  5’-CCGCAGATCTCCTCGCGGGGATGC-3’  5’-GAGGAGATCTGCGGCCGGCGTTCTGCG | pJAM1409  pTA1515  pTA1515 |
|  |  |  |
| **aRNase J:** |  |  |
| HVO_2724 NdeI | 5’-TCTCATATGGAAATCGAAATCGCAACCATAGGC-3’ | pJAM1406 |
| HVO_2724 KpnI | 5’-ATAGGTACCCTCCACCAGCTGAATCATGTTGC-3’ | pJAM1406 |
| RNJpreF_Hind | 5’-CCCAAGCTT GACGTTCATGTGCTGGTCGTAACC-3’ | pJAM4260 |
| RNJpreR_BamH | 5’-CGCGGATCC CTGCGTGCAGGTGGTCGCG-3’ | pJAM4260 |
| 2724KO_invF | 5’-ATGACTCCCGACGCGACCGAA-3’ | pJAM4261 |
| 2724KO_invR | 5’-GGTTTGTGTCTCGATATTCCGAGCAT-3’ | pJAM4261 |
| T7TerF_XbaI | 5’-CTAGTCTAGAAATAACTAGCATAACCCCTTGGGG  C-3' | pJAM4259 |
| T7TerR_Inter | 5’-TCGCGGAGGTGATGGCGTCGTCATATAGTTCCTC  CTTTCAGCAAAAAACC-3’ | pJAM4259 |
| PtnaF_Inter | 5’-GGGGTTTTTTGCTGAAAGGAGGAACTATATGACGA  CGCCATCACCTCCGC-3’ | pJAM4259 |
| PtnaR_NdeI | 5’-GGAATTCCATATGGCCCGCAATAGGTCCGCGA-3’ | pJAM4259 |
| 5FlankRNJ1F_AleI | 5’-ATCGTACACCGCGGTGGACGTTCATGTGCTGGTC  GTAAC-3’ | pJAM4259 |
| 5FlankRNJ1R_XbaI | 5’-TGCTCTAGAGGTTTGTGTCTCGATATTCCGAGC-3’ | pJAM4259 |
| RNJ1-767F | 5’-TTCGACTGCCAGGCGTAGCTCCG-3’ | HJ06 |
| RNJ1+773R | 5’-GAGGTCCGAGCCGCGCTGTTTG-3’ | HJ06 |
|  |  |  |
| **RecJ3:** |  |  |
| RecJ3F_ NdeI | 5’-TATCATATGAGCGACGAGCACGCCGGGGATTCC-3’ | pJAM1407 |
| RecJ3R_KpnI | 5’-TATGGTACCGCCGTCGTCGACAGCTCTTCGTCGA  TGTCGGCTTCGGCCATCTTCTCG-3’ | pJAM1407 |
| RecJ3F_ NdeI | 5’-TATCATATGAGCGACGAGCACGCCGGGGATTCC-3’ | pJAM4251 |
| RecJ3TAAr_XhoI | 5’-CCGCTCGAGTTACGCCGTCGTCGACAGCTCTT  CG-3’ | pJAM4251 |
| RecJ3preF_KpnI | 5’-CGGGGTACCCCTCCTGCCGTGGACCGAGGC-3’ | pJAM4255 |
| RecJ3preR_XbaI | 5’-CTAGTCTAGAGTCGCCCGACTCGATGCCGCC-3’ | pJAM4255 |
| DelRecJ3BglF | 5’- CGTTCAGATCTCAGACTGCGACCGCTCATTTTT  CG-3’ | pTA1927 |
| DelRecJ3NdeR | 5’-CGTCGCTCATATGCCCGCTAGGTCAGG-3’ | pTA1927 |
| RecJ3invF | 5’-GCCGGCGTTCGACTCTCAGACT-3’ | pJAM4256 |
| RecJ3invR | 5’-AGTCCCGCTAGGTCAGGGGGTAGT-3’ | pJAM4256 |
| RecJ3-225F | 5’-ACCCCGACCTGCTCGTCCTC-3’ | HJ01 |
| RecJ3+220R | 5’-GAGCGCCGAATCGACGTGC-3’ | HJ01 |
|  |  |  |
| **RecJ4:** |  |  |
| HVO_2889 NdeI | 5’-ATTCATATGGATTGGATTACGCACGAGGAAGAC-3’ | pJAM1405 |
| HVO_2889 KpnI | 5’-TTGGTACCAAACTGCTCGGCGGCGGCGTC-3’ | pJAM1405 |
| RecJ4preF_HindIII | 5’-CCCAAGCTTGCGCCCGGCCTCGTGTTCGC-3’ | pJAM4257 |
| RecJ4preR_BamHI | 5’-CGCGGATCCGTTCGACCTTGTCCACGGGGTGG  CC-3’ | pJAM4257 |
| RecJ4InvF | 5’-GCCGAATCCAGACTCCTCCTCG-3’ | pJAM4258 |
| RecJ4InvR  DelRecJ4BamF  DelRecJ4NdeR | 5’-TGCGTTGAGCAAACAACCCCTAAC-3’  5’-AGCCGGATCCAGACTCCTCCTCGCGCGG-3’  5’-CCAATCCATATGGTTGAGCAAACAACCCCTAAC  TAAAACGATTGTCG-3’ | pJAM4258  pTA1894  pTA1894 |
| RecJ4-861F | 5’-CCACACGGAACCAGCGACTCAT-3’ | HJ03 |
| RecJ4+801R | 5’-TGTCGTTCGTCTCGTTTTCCGC-3’ | HJ03 |
|  |  |  |
| **RecJ3/aRNase J:** | |  |
| Pfdx_BlpF | 5’-TAGTATCGCTGAGCCCGTGGATAAAACCCCTCG  TTG-3’ | pJAM4252 |
| Pfdx_InterR | 5’-TGGTTGCGATTTCGATTTCCATCACTGCAGAGTTC  GGCTTCCG-3’ | pJAM4252 |
| Pfdx_InterF | 5’-CGGAAGCCGAACTCTGCAGTGATGGAAATCGAAAT  CGCAACCA-3’ | pJAM4252 |
| StrepRNJ1_BlpR | 5’-TAGTATCGCTCAGCTCACTTCTCGAACTGCGGG  TG-3’ | pJAM4252 |
|  |  |  |
| DNA and RNA substrates | 5’-6-FAM-TTCGGCGACTGATGTTGATTGGC-3’ (5’D)  5’-TTCGGCGACTGATGTTGATTGGC-6-FAM-3’ (3’D)  5’-6-FAM-UUCGGCGACUGAUGUUGAUUGGC-3’ (5’R)  5’-UUCGGCGACUGAUGUUGAUUGGC-6-FAM-3’ (3’R)  5’ 6-FAM-CGAACUGCCUGGAAUCC*U*G*U*CGAACUGUAG-3’ (5’30*R) | Substrates and M_r_ standards are 6-FAM labeled at 5’-end or 3’-end as indicated. |
| DNA and RNA  M_r_ standards | 5’ 6-FAM TTCGG-3’  5’ 6-FAM TTCGGCGACT-3’  5’ 6-FAM UUCGG-3’  5’ 6-FAM UUCGGCGACU-3’ |  |
| DNA and RNA unlabeled | 5’-TTCGGCGACTGATGTTGATTGGC-3’  5’-UUCGGCGACUGAUGUUGAUUGGC-3’ |  |
|  |  |  |

**Abbreviations:** *flag-*, N-terminal Flag tag (MDYKDDDDK-); *flag-his6-*, N-terminal Flag-His6-tag with a GT linker (MDYKDDDDKGTHHHHHH-); *his6*-, N-terminal His6 thrombin cleavage tag (MGSSHHHHHHSSGLVPRGSHM-); -*strepII*, C-terminal StrepII tag with GT linker (-GTWSHPQFEK); *cdc48a*, *hvo_2380*; *samp1*, *hvo_2619*; *samp2*, *hvo_0202*; *samp3*, *hvo_2177*; *rnj*, *hvo_2724*; *recJ4*, *hvo_2889*; *recJ3*, *hvo_1018*; *p2.rrn*, ribosomal RNA P2 promoter of *Halobacterium salinarium*; *p.fdx*, *H. volcanii* ferredoxin promoter; *p.tnaA*, tryptophan-inducible promoter; *pitA_Nph_*, *Natronomonas pharaonis pitA* (*np_2262A*); Ap^r^ (ampicillin), Nv^r^ (novobiocin) and Km^r^ (kanamycin) resistance; *mrr*, Mrr (modified DNA rejection and restriction) family endonuclease (HVO_0682); T7term, T7 transcriptional terminator. Underlined oligonulceotide sequence refers to restriction modification sites. M_r_, molecular mass standards; 6-FAM, 6-carboxyfluorescein; *, phosphorothioate modified.

**Supplemental References**

1. Wendoloski D, Ferrer C, Dyall-Smith ML. 2001. A new simvastatin (mevinolin)-resistance marker from *Haloarcula hispanica* and a new *Haloferax volcanii* strain cured of plasmid pHV2. Microbiology 147:959-64.

2. Allers T, Ngo HP, Mevarech M, Lloyd RG. 2004. Development of additional selectable markers for the halophilic archaeon *Haloferax volcanii* based on the *leuB* and *trpA* genes. Appl Environ Microbiol 70:943-53.

3. Allers T, Barak S, Liddell S, Wardell K, Mevarech M. 2010. Improved strains and plasmid vectors for conditional overexpression of His-tagged proteins in *Haloferax volcanii*. Appl Environ Microbiol 76:1759-69.

4. Miranda H, Nembhard N, Su D, Hepowit N, Krause D, Pritz J, Phillips C, Söll D, Maupin-Furlow J. 2011. E1- and ubiquitin-like proteins provide a direct link between protein conjugation and sulfur transfer in archaea. Proc Natl Acad Sci U S A 108:4417-22.

5. Hepowit NL, de Vera IM, Cao S, Fu X, Wu Y, Uthandi S, Chavarria NE, Englert M, Su D, Sӧll D, Kojetin DJ, Maupin-Furlow JA. 2016. Mechanistic insight into protein modification and sulfur mobilization activities of noncanonical E1 and associated ubiquitin-like proteins of Archaea. FEBS J 283:3567-3586.

6. Zhou G, Kowalczyk D, Humbard M, Rohatgi S, Maupin-Furlow J. 2008. Proteasomal components required for cell growth and stress responses in the haloarchaeon *Haloferax volcanii*. J Bacteriol 190:8096-8105.

7. Humbard MA, Zhou G, Maupin-Furlow JA. 2009. The N-terminal penultimate residue of 20S proteasome α1 influences its N^α^ acetylation and protein levels as well as growth rate and stress responses of *Haloferax volcanii*. J Bacteriol 191:3794-803.

8. Prunetti L, Reuter CJ, Hepowit NL, Wu Y, Barrueto L, Miranda HV, Kelly K, Maupin-Furlow JA. 2014. Structural and biochemical properties of an extreme 'salt-loving' proteasome activating nucleotidase from the archaeon *Haloferax volcanii*. Extremophiles 18:283-93.
