# Supplementary material for: RecJ3/4-aRNase J form a Ubl-associated nuclease complex functioning in survival against DNA damage in Haloferax volcanii
Source: mBio. 2023 Jul 17;14(4):e00852-23. doi: 10.1128/mbio.00852-23 (PMC10470531; doi:10.1128/mbio.00852-23)
Supplement: Table S2 — Genome synteny of RecJ4, RecJ3, aRNase J, and Cdc48a homologs in archaea. [file mbio.00852-23-s0010.docx]

**Table S2.** Genome synteny of RecJ4, RecJ3, RNase J and Cdc48a homologs in archaea^a^.

| **General System** | **Co-occurrence**  **No. Genomes** | **Co-occurrence Partners**  **1 2** | | **Partner 2 Description (Ref.)** |  |  |
| --- | --- | --- | --- | --- | --- | --- |
| Subunits of input | 26 | Cdc48 | RecJ3 | (discussed in text) |  |  |
| Ubl-proteasomes | 264 | RecJ3 | JAMM2 | JAMM/MPN isopeptidase family protein required for Ubl-proteasome system function in *H. volcanii* (1) |  |  |
| RNA degradosomes | 179 | RNase J | Enolase | homolog of glycolytic enzyme associated with RNA degradosomes in bacteria (2, 3) |  |  |
| DNA recombination and repair | 189 | Cdc48 | RadB | homologous recombination function in *H. volcanii* (4) |  |  |
|  | 73 | RecJ4 | MutY1 | IPR011257 superfamily protein related to DNA glycosylases that act in DNA repair in eukaryotes (5) |  |  |
| Nucleotide binding and metabolism | 138 | RecJ4 | Elp3 | Elongator complex catalytic subunit homolog, modifier of the 5-position of wobble uridine tRNAs (6) |  |  |
|  | 92 | RNase J | Lsm | binds small RNAs in *H. volcanii* (7) |  |  |
|  | 261 | RNase J | GltX | glutamate-tRNA ligase (EC:6.1.1.17) |  |  |
|  | 272 | Cdc48 | PRC | PRC-barrel domain recruited biological systems including RNA processing and photosynthesis (8) |  |  |
|  | 114 | Cdc48 | NcsA | required for thiolation of wobble uridine tRNA in *H. volcanii* (9) |  |  |
|  | 279 | RecJ3 | RibL | homolog of FAD synthase that catalyzes the adenylation of FMN with ATP to produce FAD and PP_i_ (10) |  |  |
| Ribosome quality control | 97 | RecJ4 | Rqc2 | Predicted to recognize obstructed 50S subunits and promote nascent-chain proteolysis (11) |  |  |
|  | 161 | Cdc48 | CBS-RHF | Protein with Bateman domain (tandem CBS pairs) fused to ribosome hibernation promotion factor-like (RHF) domain of the IPR036567 superfamily (12) |  |  |
| Translation | 88  160  209  279  92 | RNase J  RNase J  RNase J  RNase J  RNase J | L37e  L13  S9  S2  L18e | Ribosomal proteins |  |  |
| rRNA maturation | 306 | PRC | Nob1 | endonuclease homolog associated with rRNA maturation (13) |  |  |
| Translation initiation | 268 | PRC | TibB5 | translation initiation factor IF-2 homolog (14) |  |  |
| Transcription | 207  223 | RNase J  RNase J | RpoN  RpoK | RNA polymerase subunits |  |  |
| Redox metabolism | 120 | RecJ3 | CtaB | Protoheme IX farnesyltransferase (EC:2.5.1.141) homolog |  |  |
|  | 97 | RecJ3 | CoxB1 | Cox-type terminal oxidase subunit II (EC:1.9.3.-) homolog |  |  |
|  | 93 | RecJ3 | Trx-like | Thioredoxin-like protein |  |  |
|  | 160 | Cdc48 | AldDH | Aldehyde dehydrogenase (EC 1.2.1.3) homolog |  |  |
|  | 108 | Cdc48 | SdhA | Succinate dehydrogenase (EC:1.3.5.1) subunit homologs |  |  |
|  | 101 | Cdc48 | SdhB |  |  |  |
|  | 82 | RNase J | FerB2 | Zinc-containing ferredoxin homolog |  |  |
|  | 93 | RecJ4 | FMN_red | FMN reductase/Fe-S flavoprotein homolog (15) |  |  |
| Phospholipid/ isoprenoid metabolism | 216 | RecJ3 | PLD-like | Phospholipase D/ transphosphatidylase domain protein |  |  |
|  | 543 | RNase J | IsdA1 | Bifunctional short chain isoprenyl diphosphate synthase homolog (EC:2.5.1.10, EC:2.5.1.29) |  |  |
|  | 315 | RNase J | Mvk | Mevalonate kinase (EC:2.7.1.36) |  |  |
|  | 372 | RNase J | IPK | Isopentenyl phosphate kinase (EC:2.7.4.26) |  |  |
|  | 271 | RNase J | IdiA | Isopentenyl-diphosphate delta-isomerase (EC:5.3.3.2) |  |  |
| Other/putative functions | 169 | RecJ3 | HEAT-PBS | HEAT-PBS domain protein |  |  |
|  | 114 | RecJ4 | Yip1 | DUF649 family integral membrane domain protein (IPR006977) |  |  |
|  | 112 | RNase J | DUF456 | DUF456 family protein with putative transmembrane glycine zipper |  |  |
|  | 217 | RNase J | Memo 1 | Mediator of ErbB2-driven cell motility 1 (Memo 1) homolog |  |  |
|  | 250 | Cdc48 | DUF111 | Putative nickel insertion protein |  |  |
|  | 232 | Cdc48 | Unknown function | Uncharacterized protein with predicted RNase H-like motif fold |  |  |
|  | 154 | Cdc48 | DUF58 | DUF58 family protein with Von Willebrand factor (vWF) and transmembrane (TM) domains |  |  |
|  | 227 | PRC | PGI | Probable glucose-6-phosphate isomerase |  |  |
|  | 202 | PRC | CAAX | CAAX prenyl protease 2 (IPR003675) family protein |  |  |
|  |  |  |  |  |  | |
|  |  |  |  |  |  |  |

**Table S2 References**

1. Fu X, Liu R, Sanchez I, Silva-Sanchez C, Hepowit NL, Cao S, Chen S, Maupin-Furlow J. 2016. Ubiquitin-like proteasome system represents a eukaryotic-like pathway for targeted proteolysis in archaea. mBio 7:e00379-16.

2. Chandran V, Luisi BF. 2006. Recognition of enolase in the *Escherichia coli* RNA degradosome. J Mol Biol 358:8-15.

3. Murashko ON, Lin-Chao S. 2017. *Escherichia coli* responds to environmental changes using enolasic degradosomes and stabilized DicF sRNA to alter cellular morphology. Proc Natl Acad Sci U S A 114:E8025-E8034.

4. Wardell K, Haldenby S, Jones N, Liddell S, Ngo GHP, Allers T. 2017. RadB acts in homologous recombination in the archaeon *Haloferax volcanii*, consistent with a role as recombination mediator. DNA Repair (Amst) 55:7-16.

5. Parsons JL, Elder RH. 2003. DNA N-glycosylase deficient mice: a tale of redundancy. Mutat Res 531:165-75.

6. Lin TY, Abbassi NEH, Zakrzewski K, Chramiec-Głąbik A, Jemioła-Rzemińska M, Różycki J, Glatt S. 2019. The Elongator subunit Elp3 is a non-canonical tRNA acetyltransferase. Nat Commun 10:625.

7. Fischer S, Benz J, Späth B, Maier LK, Straub J, Granzow M, Raabe M, Urlaub H, Hoffmann J, Brutschy B, Allers T, Soppa J, Marchfelder A. 2010. The archaeal Lsm protein binds to small RNAs. J Biol Chem 285:34429-38.

8. Anantharaman V, Aravind L. 2002. The PRC-barrel: a widespread, conserved domain shared by photosynthetic reaction center subunits and proteins of RNA metabolism. Genome Biol 3:RESEARCH0061.

9. Chavarria NE, Hwang S, Cao S, Fu X, Holman M, Elbanna D, Rodriguez S, Arrington D, Englert M, Uthandi S, Söll D, Maupin-Furlow JA. 2014. Archaeal Tuc1/Ncs6 homolog required for wobble uridine tRNA thiolation is associated with ubiquitin-proteasome, translation, and RNA processing system homologs. PLoS One 9:e99104.

10. Mashhadi Z, Xu H, Grochowski LL, White RH. 2010. Archaeal RibL: a new FAD synthetase that is air sensitive. Biochemistry 49:8748-55.

11. Lytvynenko I, Paternoga H, Thrun A, Balke A, Müller TA, Chiang CH, Nagler K, Tsaprailis G, Anders S, Bischofs I, Maupin-Furlow JA, Spahn CMT, Joazeiro CAP. 2019. Alanine tails signal proteolysis in bacterial ribosome-associated quality control. Cell 178:76-90.e22.

12. Ueta M, Ohniwa RL, Yoshida H, Maki Y, Wada C, Wada A. 2008. Role of HPF (hibernation promoting factor) in translational activity in *Escherichia coli*. J Biochem 143:425-33.

13. Veith T, Martin R, Wurm JP, Weis BL, Duchardt-Ferner E, Safferthal C, Hennig R, Mirus O, Bohnsack MT, Wöhnert J, Schleiff E. 2012. Structural and functional analysis of the archaeal endonuclease Nob1. Nucleic Acids Res 40:3259-74.

14. Gäbel K, Schmitt J, Schulz S, Näther DJ, Soppa J. 2013. A comprehensive analysis of the importance of translation initiation factors for *Haloferax volcanii* applying deletion and conditional depletion mutants. PLoS One 8:e77188.

15. Zhao T, Cruz F, Ferry JG. 2001. Iron-sulfur flavoprotein (Isf) from *Methanosarcina thermophila* is the prototype of a widely distributed family. J Bacteriol 183:6225-33.
